# A 7-gene expression signature predicts immune microenvironment remodeling and neoadjuvant chemo-immunotherapy response in lung squamous cell carcinoma

**DOI:** 10.3389/fimmu.2026.1696792

**Published:** 2026-02-26

**Authors:** Shaoling Li, Junhong Guo, Yan Huang, Zhengwei Dong, Ranyue Wang, Huifang Liu, Yaoqi Xiao, Jin Wang, Xing Li, Zhan Huang, Tao Hu, Changbin Zhu, Likun Hou, Chunyan Wu

**Affiliations:** 1 Department of Pathology, Shanghai Pulmonary Hospital, School of Medicine, Tongji University, Shanghai, China; 2 Nanchang University, Nanchang, China; 3 Department of Medicine, Amoy Diagnostics Co., Ltd., Xiamen, China

**Keywords:** *HOXC13*, lung squamous cell carcinoma, neoadjuvant chemo-immunotherapy, RNA sequencing, tumor microenvironment

## Abstract

**Background:**

Neoadjuvant chemo-immunotherapy (NCI) has significantly improved outcomes in advanced lung squamous cell carcinoma (LUSC). However, some patients remain resistant to NCI, resulting in poor outcomes. The mechanisms behind this resistance remain unclear.

**Methods:**

Forty LUSC patients receiving NCI were selected and categorized into major pathological response (MPR) and non-MPR groups based on their pathological response. Pre- and post-treatment samples underwent bulk RNA sequencing (RNA-seq) to assess the composition of immune cell subtypes, including T cells, B cells, NK cells, dendritic cells, and macrophages. A panel of 134 immune cell subtypes were further analyzed to differentiate between “cold” and “hot” tumor immune phenotypes.

**Results:**

After surgery, 75% of patients achieved MPR, while 25% were classified as non-MPR. In MPR patients, NCI transformed the tumor immune microenvironment (TME) from a “cold” to a “hot” phenotype, characterized by increased anti-tumor immune activity. We identified seven genes potentially linked to NCI response. Among these, *HOXC13* was associated with reduced immune-cell infiltration and inferior NCI response. High *HOXC13* expression was associated with worse progression-free survival (PFS) and overall survival (OS), as confirmed by the OAK database.

**Conclusions:**

NCI altered the TME and was linked to treatment response in LUSC. Identifying predictors of immunotherapy efficacy, such as *HOXC13*, provides potential strategies to overcome resistance in clinical practice.

## Introduction

Lung cancer is the leading cause of cancer-related mortality globally, accounting for 18% of all diagnosed cancers (1, 2). Lung squamous cell carcinoma (LUSC) is the second most common type, responsible for 20% to 30% of lung cancer-related deaths (3, 4). As a subtype of non-small cell lung cancer (NSCLC), LUSC includes spindle cell carcinoma (5), and is characterized by a fish-scale-like cell surface. It occurs predominantly in older men and is strongly associated with smoking (6).

For potentially resectable LUSC, surgery remains the cornerstone of treatment; however, for patients with intermediate to advanced LUSC, the surgical resection rate is low (~50%), leading to early postoperative recurrence (7, 8). Given the relative paucity of actionable oncogenic alterations in LUSC (9), opportunities for neoadjuvant targeted therapy are limited. Consequently, neoadjuvant chemo-immunotherapy (NCI) has become the predominant preoperative strategy (10, 11). Immune checkpoint inhibitors (ICIs) targeting programmed cell death protein 1 (PD-1) and its ligand PD-L1 are commonly used in immunotherapy, with favorable response observed in approximately 20% of lung cancer patients (12, 13). NCI combined with surgical treatment has shown a high radical resection rate (87.0%) for locally advanced LUSC, with pathological complete response (pCR) achieved in 26.1% of patients (14). Preclinical studies have demonstrated that ICIs combined with chemotherapy can further enhance the host immune response and inhibit cancer cells immune escape (15), underscoring the clinical promise of NCI. A common indicator of NCI efficacy is the “major pathological response” (MPR), defined as no more than 10% residual viable tumor cells in histopathological sections after treatment (16). Despite these advances, the average MPR rate for neoadjuvant ICIs is approximately 32% (ranging from 18% to 63%) (17), indicating that most patients exhibit resistance (non-MPR), the underlying mechanisms of which remain poorly defined.

In contrast to chemotherapy and targeted therapy, immunotherapy primarily targets the immune microenvironment rather than the tumor cells directly. Immunotherapy has the potential to reverse the tumor’s immunosuppressive microenvironment, trigger the body’s immune response, and ultimately eradicate the tumor (18, 19). Previous case reports have shown that NSCLC patients receiving neoadjuvant immunochemotherapy with MPR exhibited a large number of infiltrating immune cells in tumor tissues, including cytotoxic CD8^+^ T cells, monocyte-derived dendritic cells, and macrophages (20). Hu et al. demonstrated by bulk RNA-seq that neoadjuvant NSCLC tumors harbor an immunosuppressive microenvironment, which immuno-chemotherapy subsequently remodels by promoting the expansion and activation of cytotoxic CD8^+^T cells and CD16^+^NK cells (21). These studies suggest that immunotherapy can convert tumors from an immune “cold” to a “hot” phenotype. However, most investigations of the NSCLC/LUSC tumor immune microenvironment have been cross-sectional or have relied predominantly on post-operative specimens, limiting resolution of how NCI reshapes tumor-immune states from pre- to post-treatment (22). Moreover, in LUSC-where NCI is the prevailing neoadjuvant approach-clinically deployable pre-treatment biomarkers capable of prospectively identifying non-responders remain lacking.

To address these unmet needs, we assembled a cohort of resectable LUSC with paired pre- and post-NCI tumor samples and performed bulk RNA sequencing (RNA-seq) to quantify immune-cell enrichment and phenotypic shifts using signature-based deconvolution. We then applied weighted gene co-expression network analysis (WGCNA) to identify genes/modules associated with NCI efficacy. Together, these analyses delineate dynamic immunologic remodeling across treatment and support an immune-microenvironment-driven, preoperative predictor of NCI response tailored to LUSC.

## Methods

**Figure 1:**
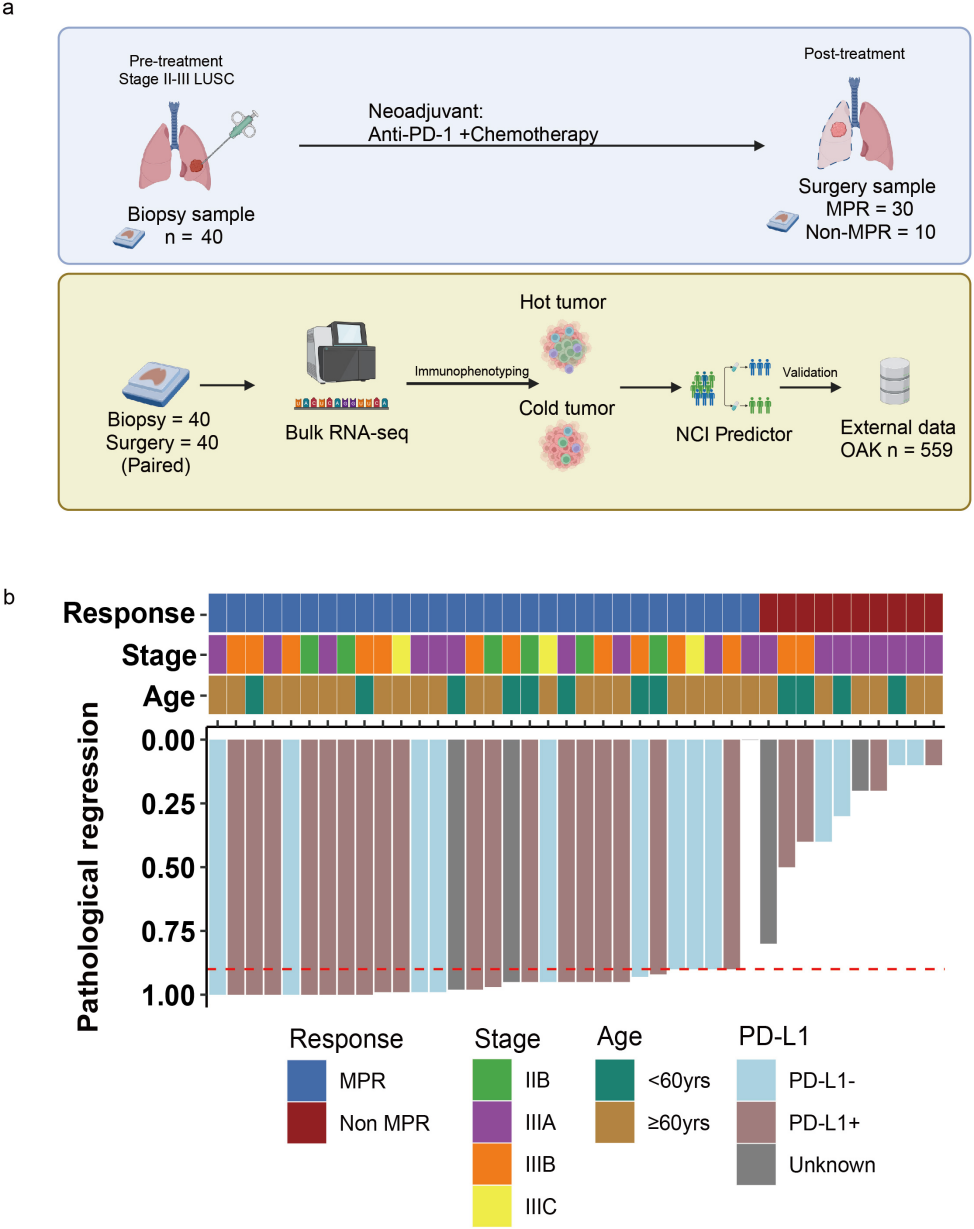
Study design baseline characteristics. **(a)** Experimental design process. **(b)** Representative patient characteristics.

The study design is shown in **Figure 1**. A brief description is given below.

### Ethics approval and consent to participate

The study protocol was approved by the Institutional Review Board of Tongji University affiliated Shanghai Pulmonary Hospital (IRB: K24-084Y), and all participants provided written informed consent.

### Study population

This retrospective study consecutive enrolled 40 patients with LUSC who received NCI at Shanghai Pulmonary Hospital between January 7, 2020, and September 7, 2022. The key inclusion criteria were: (1) clinical stage IIB-IIIC LUSC diagnosed by imaging and pathology; (2) no prior systemic chemotherapy, radiotherapy, surgery, or targeted therapies; and (3) absence of *EGFR*, *ALK*, and other driver gene mutations, with normal organ function. The main exclusion criteria were inoperable tumors after treatment or as a result of clinical incident, immunodeficiency, current systemic immunosuppressive therapy, and active autoimmune or infectious diseases. Additionally, patients lacking paired pre- and post-treatment tissue samples were also excluded.

Patients received 2 to 4 cycles of NCI therapy (PD-1 antibody + chemotherapy) followed by surgery. The study flow chart is depicted in **Figure 1**. Additionally, we utilized transcriptomic data of patients from the OAK Clinical Trial (NCT02008227) to validate the impact of relevant genes on the efficacy of immunotherapy in lung cancer patients.

### Sample collection

Pre-NCI treatment primary tumor tissues were obtained via percutaneous pulmonary, bronchoscopy, or EBUS-guided biopsies. Post-treatment tissues were collected during surgical resection. A total of 40 paired samples were collected for bulk RNA-seq. All specimens were prepared into formalin-fixed and paraffin-embedded (FFPE) blocks by pathologists and stored at room temperature for subsequent analysis.

### RNA-seq library construction and sequencing

RNA was extracted from FFPE tissue samples (n = 80) using an FFPE RNA Extraction kit (Amoy Diagnostics, Xiamen, China). Oligo(dT) magnetic beads were used to enrich mRNAs with polyA tails in total RNA, and the fragments were then broken up in a fragmentation buffer that contained divalent cations. Using random hexamer primers and the fragmented mRNA as a template, first- and second-strand cDNAs were synthesized, followed by end repair, poly-A 3’ ends, and splice sites. DNA fragment enrichment, and the NEBNext^®^ Ultra™ RNA Library Prep Kit for Illumina^®^ (Illumina, San Diego, CA, USA) were utilized to create the RNA library to build RNA-seq collections. Library quality was assessed on an Agilent Bioanalyzer 2100 system (Agilent Technologies, Palo Alto, CA, USA). Only libraries with an RNA integrity number (RIN) ≥ 6.0 and concentration ≥ 10 ng/µL were processed further. Finally, sequence analysis of RNA-Seq data was performed using the Illumina NovaSeq 6000 platform for the 150-bp end-to-end model. For each sample, a minimum of 10 Gb of clean data was generated, ensuring sufficient coverage for transcriptome profiling and downstream analyses.

### Read processing, normalization, batch correction and differential expression

The number of read segments mapped to each gene was calculated using FeatureCounts (1.5.0-p3). The FPKM for each gene was then calculated based on gene length and the number of read segments mapped to that gene was calculated. Raw counts were converted to transcripts per million (TPM), and then transformed to log_2_ (TPM + 1) for normalization. Samples with more than 50% of the genes having zero values were excluded. For gene-level differential expression between paired pre- and post-treatment tumors, per-gene differences were assessed using the Wilcoxon signed-rank test (23). Statistical significance was defined as two-sided p<0.05. Given the exploratory and small-sample nature of this study, p-values are reported as nominal alongside effect-size descriptors. All analyses were performed in R (v4.1.0).

### Cell type annotation and cluster marker identification

The CIBERSORT algorithm (v1.03) with the LM22 signature matrix was applied to estimate the abundance of immune cell expansion for each sample based on the gene expression file. Immune-infiltration scores of bulk-RNA-seq data were estimated using ssGSEA. The subtypes of tumor immune microenvironment (TME) related cells based on previous studies (24).Immune-infiltration scores were further quantified by ssGSEA using two complementary reference sets—(1) the 29 immune-associated hallmarks from published signatures, and (2) a collection of 134 TME-related cell-type markers (T cells, B cells, NK cells, fibroblasts, dendritic cells, macrophages, etc.) derived from single-cell studies (25–30) (**Supplementary Table 2**). All ssGSEA enrichment scores were median-scaled across samples, and Louvain clustering on the 29-hallmark matrix partitioned tumors into four microenvironment subtypes: Immune-Enriched/Fibrotic (IE/F), Immune-Enriched (IE), Fibrotic (F), Depleted (D). Finally, based on their overall immune infiltration, the IE/F and IE subtypes were grouped as “hot” tumors, while the F and D subtypes were classified as “cold” tumors (24).

### Gene set enrichment analysis

Enrichment analysis was performed using the Cluster Profiler package in R(v4.1.0) (31). The gene set c2.cp.kegg.v7.0.symbols from Molecular Signatures Database (MSigDB) was used for GSEA analysis, and KEGG pathways were screened for significant enrichment with nominal two-sided P value < 0.05.

### Weighted gene co-expression network analysis

WGCNA was performed to identify gene modules associated with “cold” and “hot” tumors in the immune microenvironment. Gene expression data (log_2_(TPM + 1)) were preprocessed by removing low-variance genes and then quantile-normalized across samples to reduce noise and standardize data distribution. WGCNA was carried out using the “WGCNA” R package.

The network was constructed using a soft-thresholding parameter (β), determined by the lowest power value that resulted in a scale-free topology fit of R²= 0.9. A clustering dendrogram was generated to visualize gene modules, with dynamic module detection performed using the dynamic tree cut method. Modules with high similarity were merged at a mergeCutHeight of 0.20. The minimum module size was set to 30 genes (minModuleSize = 30) to avoid fragmenting smaller but biologically relevant gene clusters. The soft-threshold power β was selected as 4. To identify the modules most strongly associated with immune infiltration scores, Pearson correlation analyses were performed between the module eigengenes (MEs) and “Cold” tumors. The modules with the highest correlation to “Cold” tumors were selected for further analysis.

### Statistical analysis

Immune-cell enrichment and pathway/signature scores were compared using Wilcoxon tests: Wilcoxon signed-rank for paired pre- *vs* post-treatment comparisons within patients, and Wilcoxon rank-sum for between-group comparisons (MPR *vs* non-MPR). Overall survival (OS) and progression-free survival (PFS) were estimated by the Kaplan–Meier method; group differences were evaluated using the log-rank test. Hazard ratios (HRs) with 95% confidence intervals were obtained from Cox proportional-hazards models (univariable unless otherwise specified). Unless noted otherwise, statistical significance was defined as two-sided p<0.05. Analyses were conducted in R (v4.1.0) using the survival and survminer packages.

## Results

### Patient characteristics

Between January 7, 2020, and September 7, 2022, 40 eligible patients signed informed consent and were enrolled (**Table 1**). The median age was 67 years (range 50-76); 97.5% (n = 39) were male, and 2.5% (n = 1) was female. At baseline, 6 patients (15%) had stage IIB disease according to the AJCC^8th^ edition, while 34 patients (85%) were classified as stage III (including IIIA, IIIB, and IIIC). Subsequently, all patients received NCI therapy (PD-1 antibody plus chemotherapy) followed by surgical resection (**Figure 1a**). Based on postoperative pathological evaluation, patients were categorized into non-MPR (10 patients, 25%), and MPR (30 patients, 75%). Selected patient characteristics are shown in **Figure 1b**.

**Table 1:** The patient characteristics.

Characteristics	No. of patients (%)
Age, years, median [range]	67 [50-76]
Sex, n [%]	
Male	39 [97.5%]
Female	1 [2.5%]
Stage, n [%]	
IIB	6 [15%]
IIIA	18 [45%]
IIIB	13 [32.5%]
IIIC	3 [7.5%]
Response, n [%]	
Non MPR	10 [25%]
MPR	30 [75%]

### Significantly increased immune infiltration in MPR patients post-NCI

**Figure 2:**
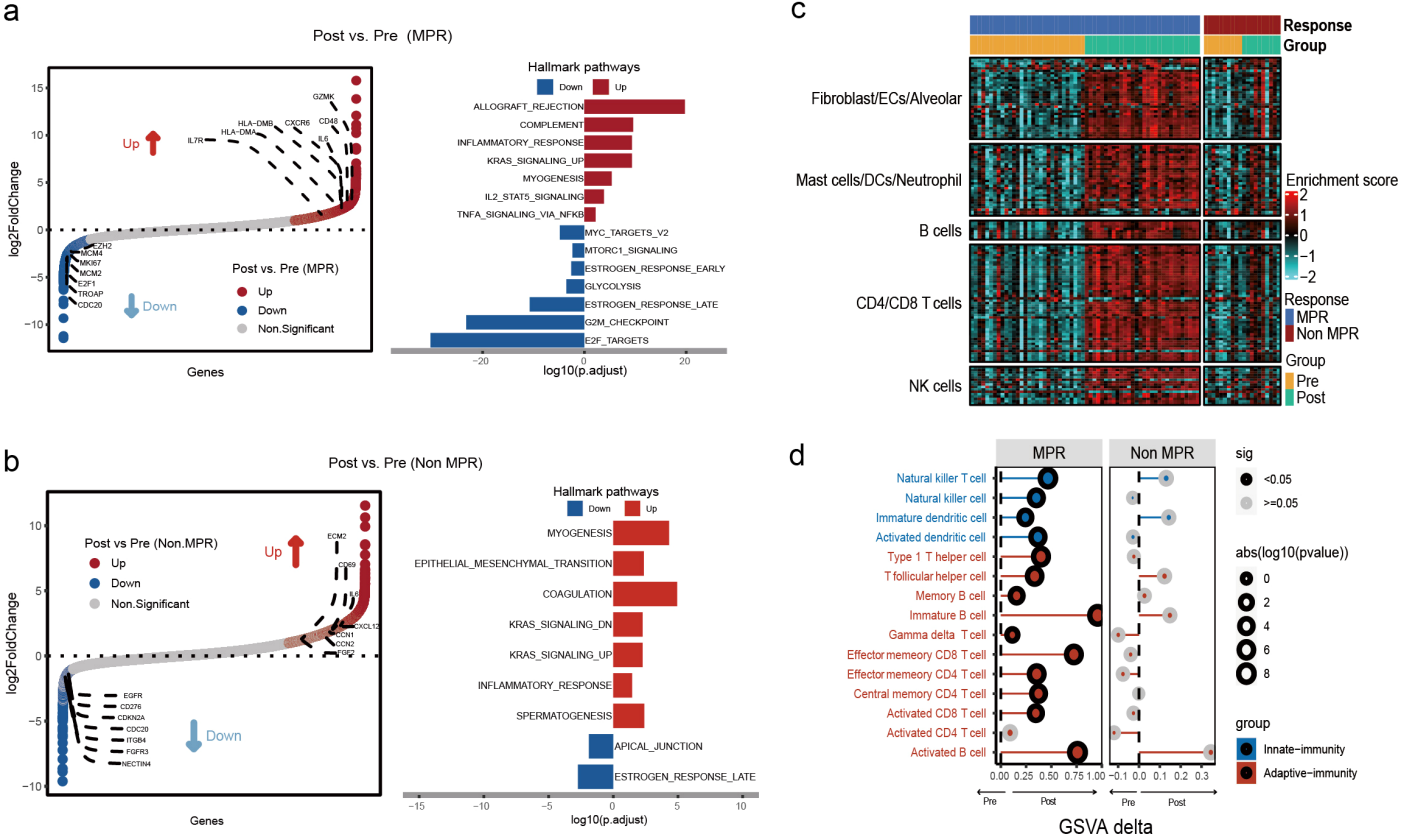
Patients in the MPR group showed a significant increase in immune cell infiltration post-NCI. **(a)** Hallmark pathways of differentially expressed genes pre- and post-NCI in MPR patients. **(b)** Hallmark pathways of differentially expressed genes pre- and post-NCI in non-MPR patients. **(c)** Heatmap of enrichment scores for 134 immune cell subtypes. **(d)** Match plot demonstrating 15 immune cell infiltrations in LUSC patients pre- and post-NCI. Patients were divided into MPR and non-MPR according to pathological response. Blue means innate immunity, red means adaptive immunity.

Transcriptome sequencing was performed on tumor samples from MPR and non-MPR groups pre- and post-NCI. Signaling pathway enrichment analysis was subsequently performed on the upregulated gene expression pre- and post-treatment in the MPR and non-MPR groups (|log_2_ fold-change|≥ log_2_(1.5) & p<0.05). In the MPR group, various pathways associated with immune response and anti-tumor effects showed significant upregulation post-treatment, including allograft rejection, complement system, and inflammatory response (**Figure 2b**). Downregulated genes were mainly enriched in oncogenic pathways such as, MYC, mTORC1, and glycolysis (**Figures 2a, b**). These results suggest that NCI activated multiple important immune and antitumor pathways, enhancing tumor immune responses in patients with MPR. Conversely, pathway enrichment analysis of patients in the non-MPR group pre- and post-treatment indicated fewer upregulated pathways. These pathways mainly involved myogenesis, epithelial-mesenchymal transition, and coagulation pathways (**Figure 2b**). Notably, gene sets annotated to inflammatory response were less prominently represented among upregulated genes in the non-MPR group than in the MPR group, although a modest enrichment signal remained detectable post-NCI (**Supplementary Figures S1a, b**). To contextualize these patterns, we computed ssGSEA enrichment scores for a panel of 134 immune cell type signature gene sets curated from six single-cell studies and projected them onto bulk RNA-seq, organizing columns by timepoint (pre *vs* post NCI) and response (MPR *vs* non-MPR) to visualize group-level shifts (**Figure 2c**; **Supplementary Table 1**). In the MPR group, multiple immune-cell-related signatures increased after NCI, whereas such changes were limited in non-MPR.

We next quantified immune-cell enrichment at the patient level. In MPR, enrichment increased after NCI for immature B cells (p<0.01), activated B cells (p<0.01), effector-memory CD8+T cells (p<0.01), and natural killer T cells (p<0.01) (**Figure 2d**). In non-MPR, no significant pre/post changes were observed across common immune cell (**Figure 2d**). At baseline, immune-cell enrichment did not differ between MPR and Non MPR group (**Supplementary Figure S1c**). Post-NCI, MPR tumors displayed higher antitumor immune activity than non-MPR, including activated B cells (p<0.01), activated CD8+T cells (p=0.02), activated dendritic cells (p<0.01), and central-memory CD4+ T cells (p<0.01) (**Supplementary Figure S1d**).

Collectively, these findings indicate that NCI elicits robust adaptive immune activation in responders, while immune remodeling is blunted in non-responders, aligning with their inferior pathological response.

### Immune microenvironment clustering based on 134 single-cell features distinguishes “hot” and “cold” tumors associated with therapeutic response

**Figure 3:**
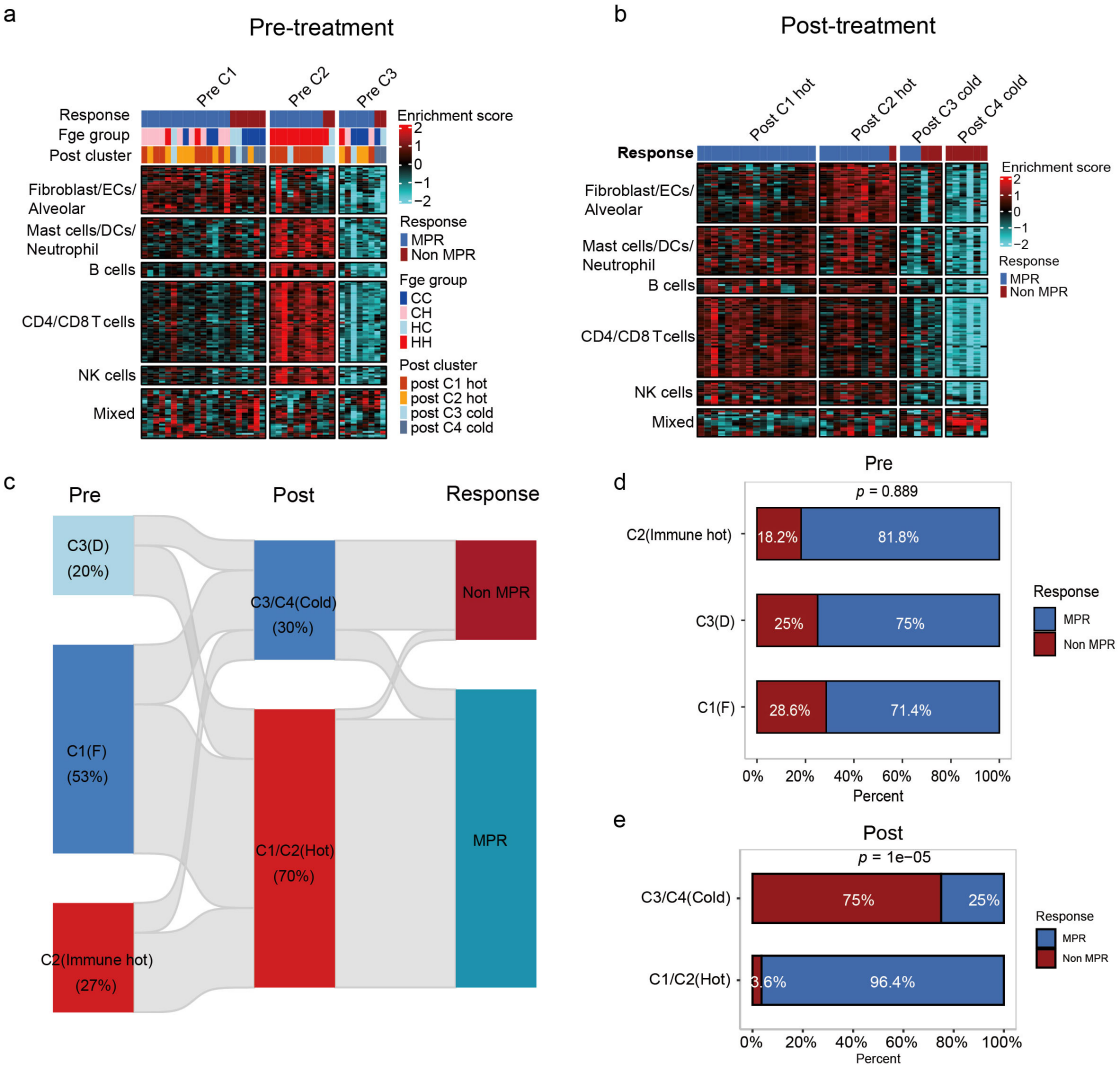
Immune profiling identifies a subset of immune hot tumors with favorable outcomes from NCI. **(a)** The heatmap demonstrated the immune cell enrichment scores for the baseline sample (pre-treatment). Clustering based on immune cell enrichment in the baseline samples revealed that the baseline samples can be categorized into 3 clusters (cluster 1, cluster 2, and cluster 3). These immune cells can be divided into 6 major categories: Fibroblast/ECs/Alveolar, Mast cells/DCs/Neutrophil, B cells, CD4/CD8 T cells, NK cells and Mixed. **(b)** The heatmap demonstrated the immune cell enrichment scores of the surgical samples (post-treatment). Clustering the samples according to immune cell enrichment revealed that the baseline samples could be divided into 4 clusters (cluster 1, cluster 2, cluster 3 and cluster 4). These immune cells can be divided into 6 major categories: Fibroblast/ECs/Alveolar, Mast cells/DCs/Neutrophil, B cells, CD4/CD8 T cells, NK cells and Mixed. **(c)** Sankey plot demonstrating the dynamics of immune cell clusters between different treatment responses pre- and post-treatment. In the baseline samples, this C1 cluster was defined as fibrosis (F), C2 was defined as immune enrichment (Immune_hot), and C3 was defined as immune depletion (D) based on TME characteristics. In surgical samples, C1 and C2 were defined as “hot” tumors, and C3 and C4 were defined as “cold” tumors. **(d)** Distribution of MPR and non-MPR samples in clusters C1, C2 and C3 pre-treatment. **(e)** Distribution of MPR and non-MPR samples in clusters C1/C2 (hot) and C3/C4 (cold) post-treatment.

RNA-seq and immune microenvironment clustering analyses were performed on pre- and post-treatment samples to characterize “cold” and “hot” tumors in LUSC. **Figures 3a, b** illustrating immune cell enrichment scores in pre- and post-treatment samples, along with dynamic changes in immune cell composition among treatment response groups (MPR and non-MPR). The samples were categorized into three and four subgroups based on immune cell enrichment in pre-treatment and post-treatment samples respectively. According to TME characteristics, these subgroups were labeled as fibrosis (F), immune-enriched, and depleted (D) (**Supplementary Figures S2a, b**). As shown in **Figure 3b**, Cluster 1(C1) and 2 (C2) was classified as a “hot” tumor due to higher T cell and NK cell enrichment scores, while Clusters 3 (C3) and 4 (C4) were defined as “cold” tumors with lower B cell and dendritic cell enrichment scores. As for pre-treatment samples, MPR patient were predominantly concentrated in the “hot” tumor cluster, while non-MPR patients were predominantly concentrated in the “cold” tumor cluster (24) (**Figure 3a**). Post-treatment, MPR patients remained predominantly in “hot” clusters, whereas non-MPR patients persisted in “cold” clusters (**Figure 3b**; **Supplementary Figure S2b**).

According to the Sankey plot results, nearly half of the “cold” tumors converted to “hot” post-NCI (**Figure 3c**; **Supplementary Figure S2c**). Pre-treatment, MPR and non-MPR patients exhibited a balanced distribution between “cold” and “hot” tumor subpopulations (**Figure 3d**). Post-treatment, 96.4% of MPR patients were in “hot” clusters, while 75% of non-MPR patients remained in “cold” clusters (**Figure 3e**; **Supplementary Figure S2d**), underscoring the link between “hot” phenotypes and treatment response.

### Identification of 7 genes associated with the efficacy of NCI

**Figure 4:**
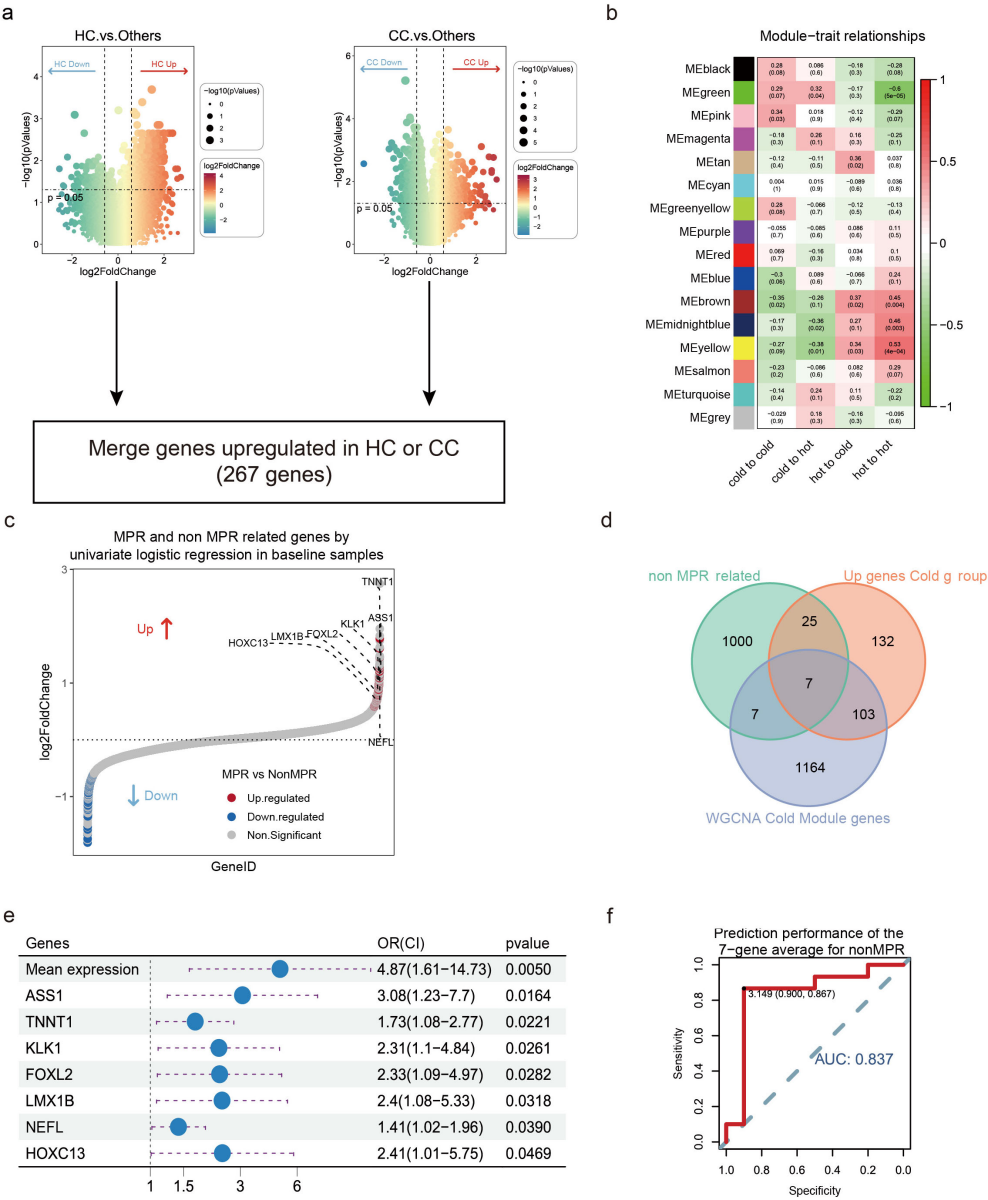
Screening for DEGs in NCI. **(a)** volcano diagram of DEGs in the cold tumor-related gene module. HC means tumors transitioning from “hot” to “cold”; CC means tumors maintaining “cold” tumor (CC). **(b)** Heatmap of module–trait correlations. Red represents positive correlations and blue represent negative correlations. **(c)** MPR and non-MPR related genes by univariate logistic regression in baseline samples. **(d)** Venn diagrams for the three datasets. The three datasets are: genes up-regulated in “cold” tumors in differential gene analysis, genes up-regulated in non-MPR, and the “cold” tumor-associated gene module mined by WGCNA. **(e)** Univariate COX regression to analyze the correlation between gene expression and treatment response. **(f)** ROC curves were used to assess the predictive performance of the 7-gene average for non-MPR.

To explore genes associated with NCI efficacy, we compared differentially expressed genes (DEGs) between tumors transitioning from “hot” to “cold” (HC) and those maintaining “cold” tumor (CC) with other subgroups, 267 genes were identified significantly upregulated in HC or CC groups (|Log2FC| > Log2 (1.5), p < 0.05) (**Figure 4a**). Furthermore, we constructed a gene co-expression network using WGCNA. Initially, we calculated Pearson correlation coefficients between all gene pairs and selected a soft-threshold power of 4 to ensure the network displayed scale-free topology. Then associated the module eigengenes with tumors transitioning from “hot” to “cold” or maintaining ‘cold’ status post-NCI therapy. The results indicated that 4 modules were significantly correlated with “cold” tumors post-NCI therapy (r > 0.3, p < 0.05) (**Figure 4b**). Additionally, using univariate logistic regression, we identified 1039 genes significantly associated with MPR (p < 0.05, **Figure 4c**). Seven genes (*TNNT1*, *FOXL2*, *LMX1B*, *NEFL*, *HOXC13*, *ASS1*, and *KLK11*) were identified as the intersection of the three datasets, defining them as genes associated with NCI efficacy (**Figure 4d**).

We conducted a detailed analysis of the expression of seven genes and immune cell infiltration pre-NCI, aiming to further explore their relationship with treatment response. Univariate Cox regression analysis demonstrated the difference of these seven genes in NCI response, revealing associations with non-MPR patients (**Figure 4e**). Receiver operating characteristic (ROC) analysis demonstrated good diagnostic efficacy for predicting non-MPR with an area under ROC curve (AUC) value of 0.837 (**Figure 4f**). Additionally, leave-one-out cross-validation confirmed that the model still exhibits strong discriminatory performance for MPR (**Supplementary Figure S3a**).

**Figure 5:**
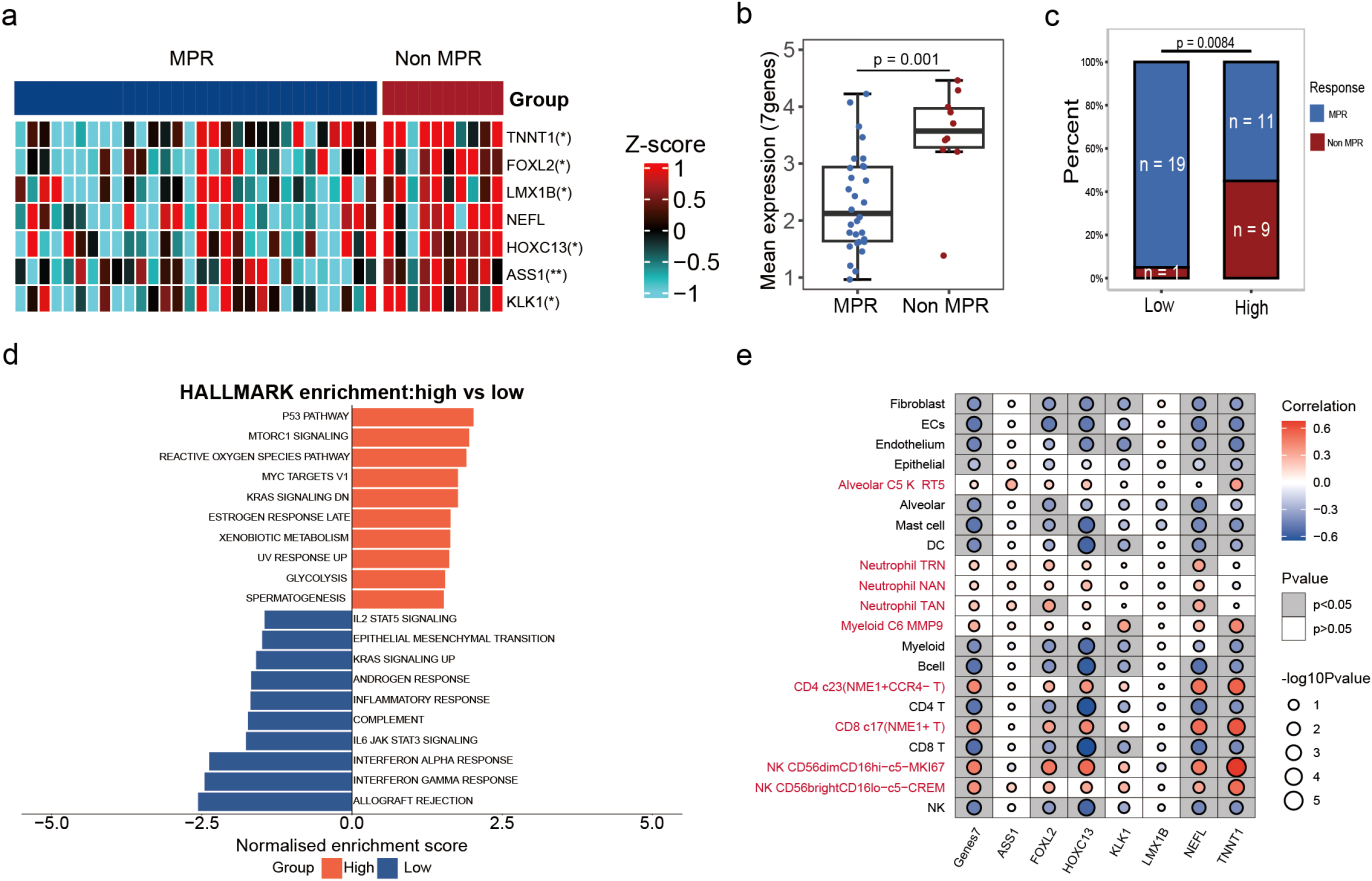
The expression of seven gene pre-treatment is associated with NCI. **(a)** Heatmap of seven genes (*TNNT1*, *FOXL2*, *LMX1B*, *NEFL*, *HOXC13*, *ASS1*, and *KLK11*) with differential expression between MPR and non-MPR groups. **(b)** Box plot of seven gene expression between MPR and non-MPR samples. **(c)** Histogram showing the distribution of high and low gene expression in MPR and non-MPR samples. **(d)** HALLMARK enrichment of DEGs and groups were categorized into high expression and low expression groups based on the expression of the seven genes. **(e)** Bubble plots demonstrating the correlation between seven specific genes and the dynamics of different immune cell types pre- and post-neoadjuvant therapy. Red represents positive correlations and blue represent negative correlations.

**Figure 5a** illustrates the expression levels of these genes in MPR and non-MPR patients, showing significantly higher expression in the non-MPR group. The mean expression of these seven genes was significantly higher in the non-MPR group compared to the MPR groups (p < 0.05, **Figure 5b**). Based on the median-defined thresholds of seven-gene expression score(cutoff=2.6), the patients were classified into high-expression and low-expression groups, only one non-MPR patient was in the low-expression group (**Figure 5c**).In multivariable analysis (adjusting for stage, age, and PD-L1 status), the seven-gene high group remained independently associated with non-MPR (OR = 3.24[1.2-12.2], p=0.038, **Supplementary Figure S3b**).In addition, in the group of seven genes with high expression, the DEGs were mainly enriched in pathways such as mTORC1 signaling, MYC targets, and KRAS signaling (**Figure 5d**). These pathways are associated with cancer oncogenic pathways. Furthermore, we analyzed the correlation between seven genes and the dynamic changes of various immune cell types pre- and post-NCI. The results indicated that the expression of these seven genes was significantly positively correlated with the CD4. c23 (NME1^+^CCR4^-^T), CD8. C17 (NME1^+^T) and CD56dimCD16hi cell type and negatively correlated with other immune cells (**Figure 5e**). Moreover, the *HOXC13* gene exhibited a positive correlation with NK cell types such as CD56dimCD16hi and CD56brightCD16lo and a negative correlation with the dynamic changes of most immune cells. The *NEFL* gene showed a negative correlation with various T cell types, such as CD8^+^T cell and CD4^+^T cell, while the *TNNT1* gene was significantly correlated with CD56dimCD16hi NK cell types (**Figure 5e**). These correlation analyses revealed the expression patterns of specific genes in various immune cell types, indicating their potential roles in the immune microenvironment.

### Validation of seven genes expression levels associated with immunotherapy for LUSC based on public databases

**Figure 6:**
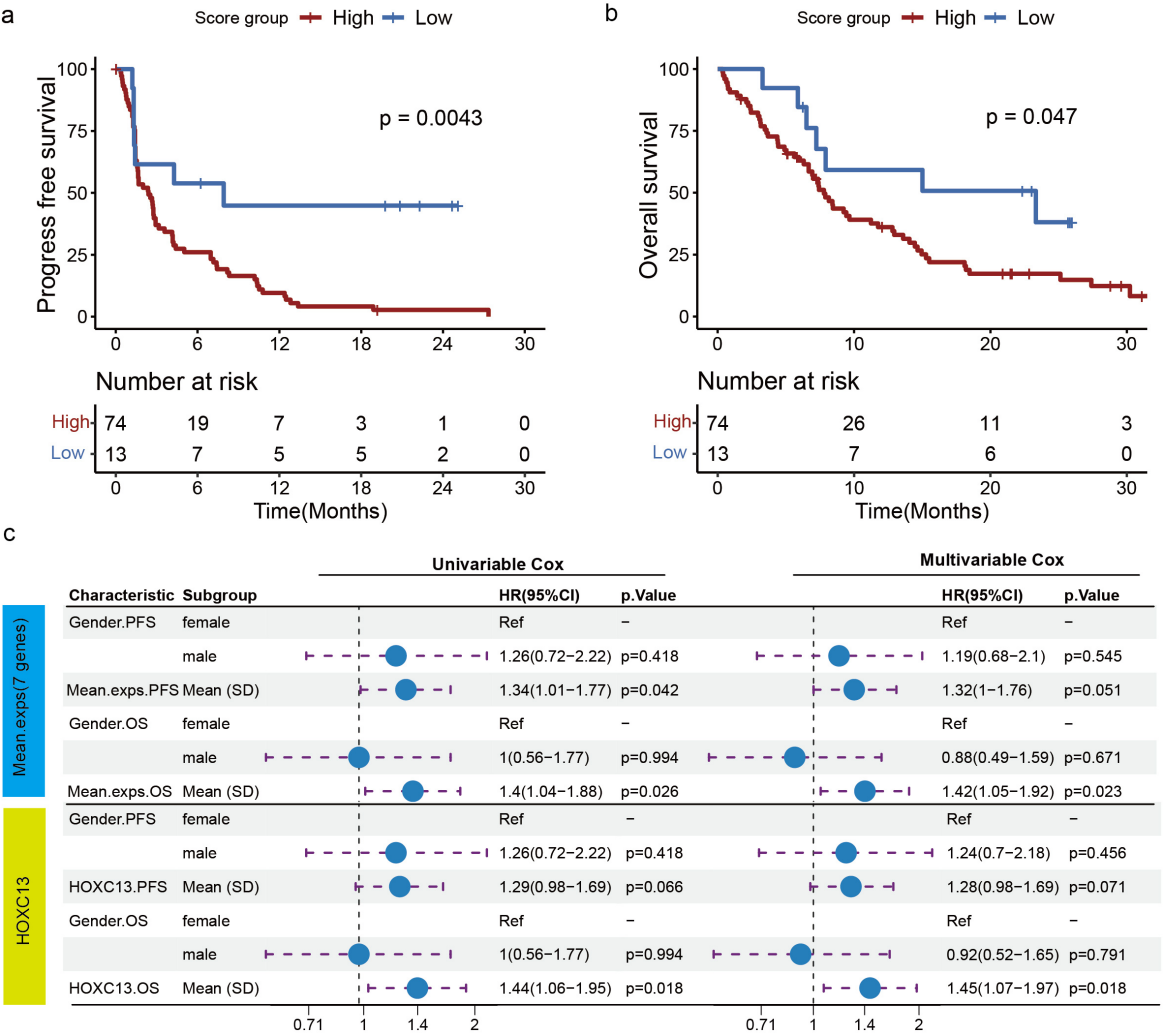
Validation of external data showing association of seven gene expression levels with immunotherapy PFS and OS in LUSC. **(a, b)** In LUSC patients receiving immunotherapy, Kaplan-Meier survival curves showed PFS **(a)** and OS **(b)** in patients with high and low 7-gene expression. **(c)** Based on the OAK database, the correlation between seven genes or *HOXC13* expression and OS and PFS in LUSC patients receiving immunotherapy was analyzed by multivariable and univariate COX regression.

In an independent neoadjuvant cohort of 10 resectable stage II–III LUSC patients (21), we defined 7-gene high expression as high risk using an optimal cutoff (cutoff = 1.5). The high-risk group showed a markedly lower MPR rate than the low-risk group (12.5% *vs* 100%, p = 0.0667) (**Supplementary Figure S3b**). Given the limited availability of public neoadjuvant LUSC datasets with pretreatment biopsies, we further assessed the signature in the phase III OAK trial. To further investigate the relationship between the expression levels of the seven genes and immunotherapy efficacy, we confirmed their correlation in a phase III clinical study (OAK trial) comparing immunotherapy versus chemotherapy in NSCLC. In the LUSC immunotherapy group, using the optimal OS-separating cut point to define 7-gene high *vs* low, patients with high gene expression had shorter PFS (p = 0.004) and OS (p = 0.047) compared to those with low gene expression (**Figures 6a, b**). However, in the LUSC chemotherapy group, the expression of the seven genes did not affect patient OS and PFS (**Supplementary Figure S4a**). These findings strongly suggest that the expression of the seven genes was associated with immunotherapy efficacy, with high expression potentially reducing its efficacy.

High expression levels of the seven genes did not impact PFS and OS between immunotherapy (MPDL3280A, Atezolizumab) and chemotherapy (Docetaxel) groups in non-LUSC and NSCLC patients (**Supplementary Figures S4b, c**). Among non-LUSC and NSCLC patients with low gene expression, there was no significant difference in PFS between immunotherapy and chemotherapy groups. Nevertheless, immunotherapy was found to significantly extend OS in NSCLC patients with low gene expression (p = 0.0034) (**Supplementary Figure S4d**). These findings suggest that the seven genes could serve as specific immunotherapeutic biomarkers in LUSC.

Furthermore, based on the OAK database, we conducted a univariate Cox regression analysis of the expressions of the seven genes with PFS and OS in NSCLC, LUSC, and LUAD patients (**Supplementary Figures S5a, b**). Furthermore, using the OAK dataset, we performed univariable Cox regression of the seven-gene expression score for PFS and OS in NSCLC, LUSC, and LUAD (**Supplementary Figures S5a, b**). Higher seven-gene expression was associated with worse OS (HR = 1.40; 95% CI, 1.04–1.88; p = 0.026) and shorter PFS (HR = 1.34; 95% CI, 1.01–1.77; p = 0.022) (**Figure 6c**). In multivariable Cox models adjusting for available baseline covariates, the associations remained directionally consistent. Notably, HOXC13 showed a significant association with shorter OS (HR = 1.44; 95% CI, 1.06–1.95; p = 0.018) and a trend toward shorter PFS (HR = 1.29; 95% CI, 0.98–1.69; p = 0.066) (**Figure 6c**).

## Discussion

Immune checkpoint inhibitors have transformed LUSC treatment, extending from metastatic disease to locally advanced, resectable tumors (32). NCI provides the opportunity for early eradication of micrometastatic lesions, thereby enhancing treatment initiation and adherence rates, and potentially eliminating live tumor cells released into the circulation during surgery (33, 34). Hence, NCI may confer additional benefits. Studies have demonstrated that NCI can significantly improve overall survival. In early-stage resectable NSCLC, NCI has achieved a 45% MPR rate (35). However, a majority of patients exhibit inadequate tumor response to immunotherapy, leading to unfavorable treatment outcomes. Therefore, this study investigated mechanisms of suboptimal NCI response in LUSC. We enrolled 40 patients with LUSC who received NCI therapy (PD-1 antibody + chemotherapy) followed by surgical resection. Encouragingly, up to 30% of resected tumors exhibited pCR or MPR, highlighting the anti-tumor efficacy of NCI in LUSC as observed in this study. This finding is consistent with previous studies reporting MPR results (35). Nevertheless, 10% of patients exhibited inadequate tumor response (non-MPR).

The TME comprises malignant cells alongside a heterogeneous mix of non-malignant stromal, vascular, and immune cell populations. With the success of immunotherapy, the role of the TME in tumor development and progression has been extensively studied (36, 37). We speculated that the low efficacy of NCI in LUSC patients could be closely related to the impaired infiltration of immune cells in the tumor. For this reason, tumor tissues pre- and post-treatment were collected for bulk RNA-seq analysis, and the enrichment of immune cells in tumor tissues was compared between the MPR group and the non-MPR group. Interestingly, we found that in the MPR group, after immunotherapy, there was a significant increase in the infiltration of immune cells in the tumor samples compared to pre-treatment. In particular, the infiltration of activated B cells, CD8^+^ T cells, NK cells and macrophages was increased. This result is similar to literature research studies (38, 39). On the one hand, Chen et al. found that in LUSC, the proportion of CD8^+^ T cell subclusters was higher in patients with MPR after immunotherapy (40). On the other hand, immune cells such as dendritic cells and macrophages were found to exhibit higher levels of anti-tumor immune responses in the immune-responsive group than in the non-immune-responsive group (41). In addition, previous studies used PD-L1 blockade to increase CD8^+^ T cell infiltration in lung cancer, and depletion of CD8^+^ T reversed its anti-cancer effect, suggesting that CD8^+^ T has anti-cancer potential (42). B cells can enhance the killing effect of CD8^+^ T lymphocytes on lung stem-like cancer cells (43). While depletion of NK cells and macrophages aggravated tumor progression (44, 45). Thus, in patients with MPR, undergoing NCI activated most of the anti-tumor immune cells to exert a significant anti-cancer effect. On the contrary, in patients with non-MPR, these immune cells were not effectively activated after treatment, resulting in ineffective NCI.

There are significant differences in the remodeling of the immune microenvironment after immunotherapy in patients with different pathological responses. We hypothesized that good responders (MPR patients) might had substantial immune cell activation, whereas poor responders (non-MPR patients) might suffer from a deficiency in the infiltration of immune cells. To further explore the immune cell typing in the samples, we classified the immune cells into subtypes based on the clustering of 134 immune cells. Pre-treatment, the samples were classified into three clusters, with cluster C1 defined as “hot” tumors and clusters C2/C3 defined as “cold” tumors based on immune cell activation. Using the same method, cells from the post-immunotherapy samples were also clustered, with the C1/C2 cluster defined as “hot” tumors and the C3/C4 cluster defined as “cold” tumors. “Hot” tumors were found to be enriched with T- and NK-cell immune cells, which have anticancer effects. In contrast, overall immune cells were not enriched in “cold” tumors. Notably, post-NCI, MPR patients tend to concentrate in the “hot” tumor subpopulation, whereas non-MPR patients concentrate in the “cold” tumor subpopulation. Previous studies have also found that tumors that respond well to immunotherapy are reprogrammed from an immune “cold” phenotype to an immune “hot” phenotype (20, 46, 47), which is consistent with the findings of this work. Patients who respond to immunotherapy exhibit robust *in vivo* activation and infiltration of effector immune cells, whereas poor responders show minimal immune cell recruitment. Consequently, strategies that enhance intratumoral immune infiltration may help overcome resistance to checkpoint blockade.

Among the seven identified genes associated with NCI response, *HOXC13* was found to be oncogenic in previous reports (48). Over expression of *HOXC13* promoted the proliferation of lung adenocarcinoma cells and facilitated cell cycle progression (49). Furthermore, in glioblastoma, the correlation between *HOXC13* expression and immune cell infiltration (50). High expression of *HOXC13* expression suppressed infiltration of γδ T cells and plasma cells (51). Interestingly, this study also found a negative correlation between *HOXC13* expression and enrichment of mostly immune cells such as T cells and B cells*. HOXC13* was associated with OS and showed a trend toward association with PFS in immunotherapy-treated LUSC, supporting *HOXC13* as a candidate marker related to immunotherapy outcomes. However, its prognostic role in LUSC appears limited, warranting further investigation. Specifically, targeting *HOXC13* expression to increase immune cell activation could potentially enhance the efficacy of NCI. Mechanistically, HOXC13 may suppress immune infiltration through several tumor-intrinsic programs. First, HOXC13 has been shown to remodel the tumor–immune microenvironment by regulating innate sensing/IFN programs (cGAS–STING–IRF3 axis) and chemokines, implying that dysregulated HOXC13 can blunt type-I IFN signaling and T-cell-supportive cytokines (51). Second, HOXC13 can reprogram epigenetics (e.g.,upregulating DNMT3A) and cell-cycle/glycolytic pathways, a milieu linked to reduced antigen visibility and non-inflamed states (52). Third, tissue data in other cancers suggest an inverse association with CD8^+^ T-cell presence, consistent with an immune-excluded phenotype (53). Collectively, these lines of evidence support a model in which HOXC13 shapes immune quality (type-I IFN tone, antigen presentation/epigenetics, chemokine milieu) rather than merely altering bulk immune-cell quantities.

High *ASS1* expression remodels the tumor microenvironment by driving a purine‐rich metabolic state that suppresses immunoproteasome activity and neoantigen presentation, resulting in diminished CD8^+^ T-cell infiltration and poor ICI response. Pharmacologic inhibition of purine synthesis in ASS1-high tumors restores a more immunogenic milieu and significantly enhances anti-PD-1 efficacy (54).*TNNT1* (55)*, FOXL2 (*56)*, LMX1B (*57)*, NEFL (*58)*, and KLK1* (59) regulate key aspects of tumor biology, including epithelial-mesenchymal transition, proliferation, metastasis, tumor grade, and extracellular matrix remodeling. These genes may indirectly influence the immune microenvironment, although direct evidence is still lacking. While their roles in tumor progression, metastasis, and prognosis in lung cancer are well-established, their specific contributions to immune microenvironment modulation remain largely unexplored.

Notably, ASS1 and LMX1B were not associated with bulk shifts in common immune-cell compartments in our data. This is compatible with mechanisms that alter immune quality (function, localization, spatial exclusion) rather than quantity per se—for example, ASS1-driven metabolic/antigen-processing effects and LMX1B-linked stromal/ECM programs that can reposition or dampen antitumor immunity without large changes in overall cell proportions (54, 60). These hypotheses call for spatial and functional validation in future cohorts.

There are some limitations of this study. First, because this study was an exploratory pilot study, the number of patients enrolled was small. Additionally, there is a notable imbalance between the MPR and non-MPR groups, which may introduce bias and affect the robustness of the findings. Therefore, a larger, well-balanced randomized controlled trial is needed to further validate our findings and mitigate these limitations. Second, the biological mechanisms underlying the association between HOXC13 expression and immunotherapy outcomes in LUSC remain unclear. In particular, whether HOXC13 contributes to an immune-excluded microenvironment and reduced immunotherapy benefit requires further functional and protein-level validation in future studies. Third, although we identified a seven-gene panel associated with response, how these genes shape the LUSC immune microenvironment is not fully defined; importantly, future studies should validate their expression at the protein level and test their association with clinical efficacy.

In conclusion, this study delineates the immune microenvironment of tumor samples from patients pre- and post-NCI. The samples were stratified into MPR and non-MPR groups based on treatment efficacy. In the MPR group, the immune microenvironment was observed to transition from an immune “cold” to an immune “hot” phenotype following NCI therapy, stimulating immune activity. *HOXC13* was identified as a key factor impairing immune cell infiltration and NCI efficacy in LUSC. This study identifies factors contributing to suboptimal NCI outcomes and suggests potential clinical targets.

## Data Availability

The original contributions presented in the study are included in the article/**Supplementary Material**. Further inquiries can be directed to the corresponding authors.

## Supplementary material

Supplementary Figure 1 Differential gene signaling pathway enrichment and tumor microenvironment differences between MPR and non MPR groups. **(a)** KEGG enrichment analysis of differentially expressed genes of MPR *vs* non-MPR samples pre-NCI. **(b)** KEGG enrichment analysis of differentially expressed genes of MPR *vs* non-MPR samples post-NCI. **(c)** Differences in enrichment of immune cells between MPR and non-MPR groups pre-NCI. **(d)** Differences in enrichment of immune cells between MPR and non-MPR groups post-NCI.

Supplementary Figure 2 Distribution of “cold” and “hot” tumors pre- and post-NCI treatment between MPR and non MPR groups. **(a)** Pre-treatment, clustering analysis of the immune microenvironment based on previously published literature was performed to define “hot” and “cold” tumors. **(b)** Post-treatment, clustering analysis of the immune microenvironment based on previously published literature was performed to define “hot” and “cold” tumors. **(c)** Sankey plot demonstrating the dynamics of “hot” and “cold” tumors between different treatment responses pre- and post-treatment. **(d)** Distribution of MPR and non-MPR samples across treatment effects. Based on the difference between hot and cold tumors pre- and post-NCI treatment, we classified treatment effects into 4 types: tumors transitioning from “hot” to “cold” (HC); maintaining “cold” tumor (CC); tumors transitioning from “cold” to “hot” (CH); and maintaining “hot” tumor (HH).

Supplementary Figure 3 Seven-gene expression performance verification. **(a)** Leave-one-out cross-validation (LOOCV) of the 7-gene score for predicting MPR; ROC curve with AUC reported. **(b)** Validation of the 7-gene signature in an independent neoadjuvant LUSC biopsy cohort (GSE207422; Junjie Hu et al., Genome Medicine, 2023). Patients (n=10) were stratified into High-risk and Low-risk groups using the optimal cutoff of the 7-gene score. **(c)** Multivariable logistic regression of the 7-gene score for MPR prediction (adjusted for stage, age, and PD-L1 status); forest plot shows adjusted odds ratios (95% CIs) and p-values.

Supplementary Figure 4 Validation of external data showing association of seven genes expression levels with chemotherapy PFS and OS in LUSC. **(a)** Based on the OAK database, the correlation between the expression of seven genes and OS and PFS in LUSC patients receiving chemotherapy was analyzed by univariate COX regression. **(b)** In LUSC patients receiving chemotherapy, Kaplan-Meier survival curves showed OS and PFS in patients with high and low gene expression. **(c)** Based on OAK database, PFS and OS were compared by Kaplan-Meier survival analysis between immunotherapy and chemotherapy groups in non-LUSC patients and whole NSCLC group.

Supplementary Figure 5 *HOXC13* expression is associated with poor outcome of LUSC immunotherapy. **(a)** Expression of seven genes in the OAK database correlates with PFS in NSCLC, LUSC, and LUAD patients by univariate COX regression analysis. **(b)** Expression of seven genes in the OAK database correlates with OS in NSCLC, LUSC, and LUAD patients by univariate COX regression analysis.

## References

[1] Kratzer TB Bandi P Freedman ND Smith RA Travis WD Jemal A . Lung cancer statistics, 2023. Cancer. (2024) 130:1330–48. doi: 10.1002/cncr.35128, PMID: 38279776

[2] Popper H . Pathologic diagnosis of lung cancer - recent developments. Curr Opin Oncol. (2024) 36:57–62. doi: 10.1097/CCO.0000000000001011, PMID: 37975321

[3] Niu Z Jin R Zhang Y Li H . Signaling pathways and targeted therapies in lung squamous cell carcinoma: mechanisms and clinical trials. Signal Transduct Target Ther. (2022) 7:353. doi: 10.1038/s41392-022-01200-x, PMID: 36198685 PMC9535022

[4] Pan Y Han H Hu H Wang H Song Y Hao Y . KMT2D deficiency drives lung squamous cell carcinoma and hypersensitivity to RTK-RAS inhibition. Cancer Cell. (2023) 41:88–105.e8. doi: 10.1016/j.ccell.2022.11.015, PMID: 36525973 PMC10388706

[5] Denisov EV Schegoleva AA Gervas PA Ponomaryova AA Tashireva LA Boyarko VV . Premalignant lesions of squamous cell carcinoma of the lung: The molecular make-up and factors affecting their progression. Lung Cancer. (2019) 135:21–8. doi: 10.1016/j.lungcan.2019.07.001, PMID: 31446997

[6] Molina JR Yang P Cassivi SD Schild SE Adjei AA . Non-small cell lung cancer: epidemiology, risk factors, treatment, and survivorship. Mayo Clin Proc. (2008) 83:584–94. doi: 10.1016/S0025-6196(11)60735-0, PMID: 18452692 PMC2718421

[7] Tanaka Y Nakai T Suzuki A Kagawa Y Noritake O Taki T . Clinicopathological significance of peritumoral alveolar macrophages in patients with resected early-stage lung squamous cell carcinoma. Cancer Immunol Immunother. (2023) 72:2205–15. doi: 10.1007/s00262-023-03393-8, PMID: 36862151 PMC10992385

[8] Duan J Zhang Y Chen R Liang L Huo Y Lu S . Tumor-immune microenvironment and NRF2 associate with clinical efficacy of PD-1 blockade combined with chemotherapy in lung squamous cell carcinoma. Cell Rep Med. (2023) 4:101302. doi: 10.1016/j.xcrm.2023.101302, PMID: 38052215 PMC10772345

[9] Comprehensive genomic characterization of squamous cell lung cancers. Nature. (2012) 489:519–25. doi: 10.1038/nature11404, PMID: 22960745 PMC3466113

[10] Forde PM Spicer J Lu S Provencio M Mitsudomi T Awad MM . Neoadjuvant nivolumab plus chemotherapy in resectable lung cancer. N Engl J Med. (2022) 386:1973–85. doi: 10.1056/NEJMoa2202170, PMID: 35403841 PMC9844511

[11] Forde PM Chaft JE Smith KN Anagnostou V Cottrell TR Hellmann MD . Neoadjuvant PD-1 blockade in resectable lung cancer. N Engl J Med. (2018) 378:1976–86. doi: 10.1056/NEJMoa1716078, PMID: 29658848 PMC6223617

[12] Huai Q Luo C Song P Bie F Bai G Li Y . Peripheral blood inflammatory biomarkers dynamics reflect treatment response and predict prognosis in non-small cell lung cancer patients with neoadjuvant immunotherapy. Cancer Sci. (2023) 114:4484–98. doi: 10.1111/cas.15964, PMID: 37731264 PMC10728017

[13] Kim YH . Durvalumab after chemoradiotherapy in stage III non-small-cell lung cancer. N Engl J Med. (2019) 380:989–90. doi: 10.1056/NEJMc1900407, PMID: 30855760

[14] Xu K Yang H Ma W Fan L Sun B Wang Z . Neoadjuvant immunotherapy facilitates resection of surgically-challenging lung squamous cell cancer. J Thorac Dis. (2021) 13:6816–26. doi: 10.21037/jtd-21-1195, PMID: 35070366 PMC8743415

[15] Yang W Xing X Yeung SJ Wang S Chen W Bao Y . Neoadjuvant programmed cell death 1 blockade combined with chemotherapy for resectable esophageal squamous cell carcinoma. J Immunother Cancer. (2022) 10. doi: 10.1136/jitc-2021-003497, PMID: 35022193 PMC8756283

[16] Travis WD Dacic S Wistuba I Sholl L Adusumilli P Bubendorf L . IASLC multidisciplinary recommendations for pathologic assessment of lung cancer resection specimens after neoadjuvant therapy. J Thorac Oncol. (2020) 15:709–40. doi: 10.1016/j.jtho.2020.01.005, PMID: 32004713 PMC8173999

[17] Jia XH Xu H Geng LY Jiao M Wang WJ Jiang LL . Efficacy and safety of neoadjuvant immunotherapy in resectable nonsmall cell lung cancer: A meta-analysis. Lung Cancer. (2020) 147:143–53. doi: 10.1016/j.lungcan.2020.07.001, PMID: 32717571

[18] Xie H Shi X Wang G . Neoadjuvant immunotherapy for resectable non-small cell lung cancer. Am J Cancer Res. (2021) 11:2521–36. PMC8263648 34249414

[19] Lv B Wang Y Ma D Cheng W Liu J Yong T . Immunotherapy: reshape the tumor immune microenvironment. Front Immunol. (2022) 13:844142. doi: 10.3389/fimmu.2022.844142, PMID: 35874717 PMC9299092

[20] Jia W Zhu H Gao Q Sun J Tan F Liu Q . Case report: transformation from cold to hot tumor in a case of NSCLC neoadjuvant immunochemotherapy pseudoprogression. Front Immunol. (2021) 12:633534. doi: 10.3389/fimmu.2021.633534, PMID: 33679783 PMC7925896

[21] Hu J Zhang L Xia H Yan Y Zhu X Sun F . Tumor microenvironment remodeling after neoadjuvant immunotherapy in non-small cell lung cancer revealed by single-cell RNA sequencing. Genome Med. (2023) 15:14. doi: 10.1186/s13073-023-01164-9, PMID: 36869384 PMC9985263

[22] Liu Z Yang Z Wu J Zhang W Sun Y Zhang C . A single-cell atlas reveals immune heterogeneity in anti-PD-1-treated non-small cell lung cancer. Cell. (2025) 188:3081–96.e19. doi: 10.1016/j.cell.2025.03.018, PMID: 40147443

[23] Li Y Ge X Peng F Li W Li JJ . Exaggerated false positives by popular differential expression methods when analyzing human population samples. Genome Biol. (2022) 23:79. doi: 10.1186/s13059-022-02648-4, PMID: 35292087 PMC8922736

[24] Bagaev A Kotlov N Nomie K Svekolkin V Gafurov A Isaeva O . Conserved pan-cancer microenvironment subtypes predict response to immunotherapy. Cancer Cell. (2021) 39:845–65.e7. doi: 10.1016/j.ccell.2021.04.014, PMID: 34019806

[25] Qian J Olbrecht S Boeckx B Vos H Laoui D Etlioglu E . A pan-cancer blueprint of the heterogeneous tumor microenvironment revealed by single-cell profiling. Cell Res. (2020) 30:745–62. doi: 10.1038/s41422-020-0355-0, PMID: 32561858 PMC7608385

[26] Cheng S Li Z Gao R Xing B Gao Y Yang Y . A pan-cancer single-cell transcriptional atlas of tumor infiltrating myeloid cells. Cell. (2021) 184:792–809.e23. doi: 10.1016/j.cell.2021.01.010, PMID: 33545035

[27] Luo H Xia X Huang LB An H Cao M Kim GD . Pan-cancer single-cell analysis reveals the heterogeneity and plasticity of cancer-associated fibroblasts in the tumor microenvironment. Nat Commun. (2022) 13:6619. doi: 10.1038/s41467-022-34395-2, PMID: 36333338 PMC9636408

[28] Tang F Li J Qi L Liu D Bo Y Qin S . A pan-cancer single-cell panorama of human natural killer cells. Cell. (2023) 186:4235–51.e20. doi: 10.1016/j.cell.2023.07.034, PMID: 37607536

[29] Zheng L Qin S Si W Wang A Xing B Gao R . Pan-cancer single-cell landscape of tumor-infiltrating T cells. Science. (2021) 374:abe6474. doi: 10.1126/science.abe6474, PMID: 34914499

[30] Salcher S Sturm G Horvath L Untergasser G Kuempers C Fotakis G . High-resolution single-cell atlas reveals diversity and plasticity of tissue-resident neutrophils in non-small cell lung cancer. Cancer Cell. (2022) 40:1503–20.e8. doi: 10.1016/j.ccell.2022.10.008, PMID: 36368318 PMC9767679

[31] Yu G Wang LG Han Y He QY . clusterProfiler: an R package for comparing biological themes among gene clusters. Omics. (2012) 16:284–7. doi: 10.1089/omi.2011.0118, PMID: 22455463 PMC3339379

[32] Kang J Zhang C Zhong WZ . Neoadjuvant immunotherapy for non-small cell lung cancer: State of the art. Cancer Commun (Lond). (2021) 41:287–302. doi: 10.1002/cac2.12153, PMID: 33689225 PMC8045926

[33] McElnay P Lim E . Adjuvant or neoadjuvant chemotherapy for NSCLC. J Thorac Dis. (2014) 6 Suppl 2:S224–7. doi: 10.3978/j.issn.2072-1439.2014.04.26, PMID: 24868440 PMC4032958

[34] Topalian SL Taube JM Pardoll DM . Neoadjuvant checkpoint blockade for cancer immunotherapy. Science. (2020) 367. doi: 10.1126/science.aax0182, PMID: 32001626 PMC7789854

[35] Cloughesy TF Mochizuki AY Orpilla JR Hugo W Lee AH Davidson TB . Neoadjuvant anti-PD-1 immunotherapy promotes a survival benefit with intratumoral and systemic immune responses in recurrent glioblastoma. Nat Med. (2019) 25:477–86. doi: 10.1038/s41591-018-0337-7, PMID: 30742122 PMC6408961

[36] Shu CA Gainor JF Awad MM Chiuzan C Grigg CM Pabani A . Neoadjuvant atezolizumab and chemotherapy in patients with resectable non-small-cell lung cancer: an open-label, multicentre, single-arm, phase 2 trial. Lancet Oncol. (2020) 21:786–95. doi: 10.1016/S1470-2045(20)30140-6, PMID: 32386568

[37] Borghaei H Paz-Ares L Horn L Spigel DR Steins M Ready NE . Nivolumab versus docetaxel in advanced nonsquamous non-small-cell lung cancer. N Engl J Med. (2015) 373:1627–39. doi: 10.1056/NEJMoa1507643, PMID: 26412456 PMC5705936

[38] Casarrubios M Provencio M Nadal E Insa A Del Rosario García-Campelo M Lázaro-Quintela M . Tumor microenvironment gene expression profiles associated to complete pathological response and disease progression in resectable NSCLC patients treated with neoadjuvant chemoimmunotherapy. J Immunother Cancer. (2022) 10. doi: 10.1136/jitc-2022-005320, PMID: 36171009 PMC9528578

[39] Porciello N Gallina F Frisullo G Fusco F D'Ambrosio L Balzano V . Spatial transcriptomics highlights B cells as key contributors to a complete and durable response to chemo-immunotherapy in a patient with resectable NSCLC. J Immunother Cancer. (2025) 13. doi: 10.1136/jitc-2025-011563, PMID: 40350207 PMC12067772

[40] Chen M Ma P Zhang Y Wang D Yu Z Fu Y . Divergent tumor and immune cell reprogramming underlying immunotherapy response and immune-related adverse events in lung squamous cell carcinoma. J Immunother Cancer. (2023) 11. doi: 10.1136/jitc-2023-007305, PMID: 37857527 PMC10603341

[41] Okuda S Ohuchida K Nakamura S Tsutsumi C Hisano K Mochida Y . Neoadjuvant chemotherapy enhances anti-tumor immune response of tumor microenvironment in human esophageal squamous cell carcinoma. iScience. (2023) 26:106480. doi: 10.1016/j.isci.2023.106480, PMID: 37091252 PMC10113784

[42] Sen T Rodriguez BL Chen L Corte CMD Morikawa N Fujimoto J . Targeting DNA damage response promotes antitumor immunity through STING-mediated T-cell activation in small cell lung cancer. Cancer Discov. (2019) 9:646–61. doi: 10.1158/2159-8290.CD-18-1020, PMID: 30777870 PMC6563834

[43] Zhang X Zhang Y Xu J Wang H Zheng X Lou Y . Antigen presentation of the Oct4 and Sox2 peptides by CD154-activated B lymphocytes enhances the killing effect of cytotoxic T lymphocytes on tumor stem-like cells derived from cisplatin-resistant lung cancer cells. J Cancer. (2018) 9:367–74. doi: 10.7150/jca.20821, PMID: 29344283 PMC5771344

[44] Aktaş ON Öztürk AB Erman B Erus S Tanju S Dilege Ş . Role of natural killer cells in lung cancer. J Cancer Res Clin Oncol. (2018) 144:997–1003. doi: 10.1007/s00432-018-2635-3, PMID: 29616326 PMC11813529

[45] Wei C Yang C Wang S Shi D Zhang C Lin X . Crosstalk between cancer cells and tumor associated macrophages is required for mesenchymal circulating tumor cell-mediated colorectal cancer metastasis. Mol Cancer. (2019) 18:64. doi: 10.1186/s12943-019-0976-4, PMID: 30927925 PMC6441214

[46] Wilkins A Fontana E Nyamundanda G Ragulan C Patil Y Mansfield D . Differential and longitudinal immune gene patterns associated with reprogrammed microenvironment and viral mimicry in response to neoadjuvant radiotherapy in rectal cancer. J Immunother Cancer. (2021) 9. doi: 10.1136/jitc-2020-001717corr1, PMID: 33678606 PMC7939016

[47] Wu Y Gong X Wang K Yu C Qiu J Zhang S . Neoadjuvant endocrine therapy: A potential way to make cold hormone receptor-rich breast cancer hot. Comb Chem High Throughput Screen. (2023) 26:1030–41. doi: 10.2174/1386207325666220617145448, PMID: 35718967

[48] Dai M Song J Wang L Zhou K Shu L . HOXC13 promotes cervical cancer proliferation, invasion and Warburg effect through β-catenin/c-Myc signaling pathway. J Bioenerg Biomembr. (2021) 53:597–608. doi: 10.1007/s10863-021-09908-1, PMID: 34309767

[49] Yao Y Luo J Sun Q Xu T Sun S Chen M . HOXC13 promotes proliferation of lung adenocarcinoma via modulation of CCND1 and CCNE1. Am J Cancer Res. (2017) 7:1820–34., PMID: 28979806 PMC5622218

[50] Yu M Yu S Zhou W Yi B Liu Y . HOXC6/8/10/13 predict poor prognosis and associate with immune infiltrations in glioblastoma. Int Immunopharmacol. (2021) 101:108293. doi: 10.1016/j.intimp.2021.108293, PMID: 34763232

[51] Li M Bai G Cen Y Xie Q Chen J Chen J . Silencing HOXC13 exerts anti-prostate cancer effects by inducing DNA damage and activating cGAS/STING/IRF3 pathway. J Transl Med. (2023) 21:884. doi: 10.1186/s12967-023-04743-x, PMID: 38057852 PMC10701956

[52] Li H Gao P Chen H Zhao J Zhang X Li G . HOXC13 promotes cell proliferation, metastasis and glycolysis in breast cancer by regulating DNMT3A. Exp Ther Med. (2023) 26:439. doi: 10.3892/etm.2023.12138, PMID: 37614427 PMC10443053

[53] Dai Z Gu Z Shen R Wang J . An immunotherapy guide constructed by cGAS-STING signature for breast cancer and the biofunction validation of the pivotal gene HOXC13 via *in vitro* experiments. Front Immunol. (2025) 16:1586877. doi: 10.3389/fimmu.2025.1586877, PMID: 40861477 PMC12370726

[54] Keshet R Lee JS Adler L Iraqi M Ariav Y Lim LQJ . Targeting purine synthesis in ASS1-expressing tumors enhances the response to immune checkpoint inhibitors. Nat Cancer. (2020) 1:894–908. doi: 10.1038/s43018-020-0106-7, PMID: 35121952

[55] Ge X Du G Zhou Q Yan B Yue G. Tnnt1 Accelerates Migration . TNNT1 accelerates migration, invasion and EMT progression in lung cancer cells. Thorac Cancer. (2024) 15:1749–56. doi: 10.1111/1759-7714.15400, PMID: 38973201 PMC11320084

[56] Li J Gao L Wang A Qian H Zhu J Ji S . Forkhead box L2 is a target of miR-133b and plays an important role in the pathogenesis of non-small cell lung cancer. Cancer Med. (2023) 12:9826–42. doi: 10.1002/cam4.5746, PMID: 36846934 PMC10166978

[57] Guo X Piao H Xue Y Liu Y Zhao H . LMX1B-associated gankyrin expression predicts poor prognosis in glioma patients. J Int Med Res. (2020) 48:300060520954764. doi: 10.1177/0300060520954764, PMID: 32960116 PMC7513415

[58] Shen Z Chen B Gan X Hu W Zhong G Li H . Methylation of neurofilament light polypeptide promoter is associated with cell invasion and metastasis in NSCLC. Biochem Biophys Res Commun. (2016) 470:627–34. doi: 10.1016/j.bbrc.2016.01.094, PMID: 26801564

[59] Lenga Ma Bonda W Iochmann S Magnen M Courty Y Reverdiau P . Kallikrein-related peptidases in lung diseases. Biol Chem. (2018) 399:959–71. doi: 10.1515/hsz-2018-0114, PMID: 29604204

[60] Du W Xia X Hu F Yu J . Extracellular matrix remodeling in the tumor immunity. Front Immunol. (2023) 14:1340634. doi: 10.3389/fimmu.2023.1340634, PMID: 38332915 PMC10850336

