# Supplementary figures and images for: A 7-gene expression signature predicts immune microenvironment remodeling and neoadjuvant chemo-immunotherapy response in lung squamous cell carcinoma
Source: Front Immunol. 2026 Feb 26;17:1696792. doi: 10.3389/fimmu.2026.1696792 (PMC12979163; doi:10.3389/fimmu.2026.1696792)

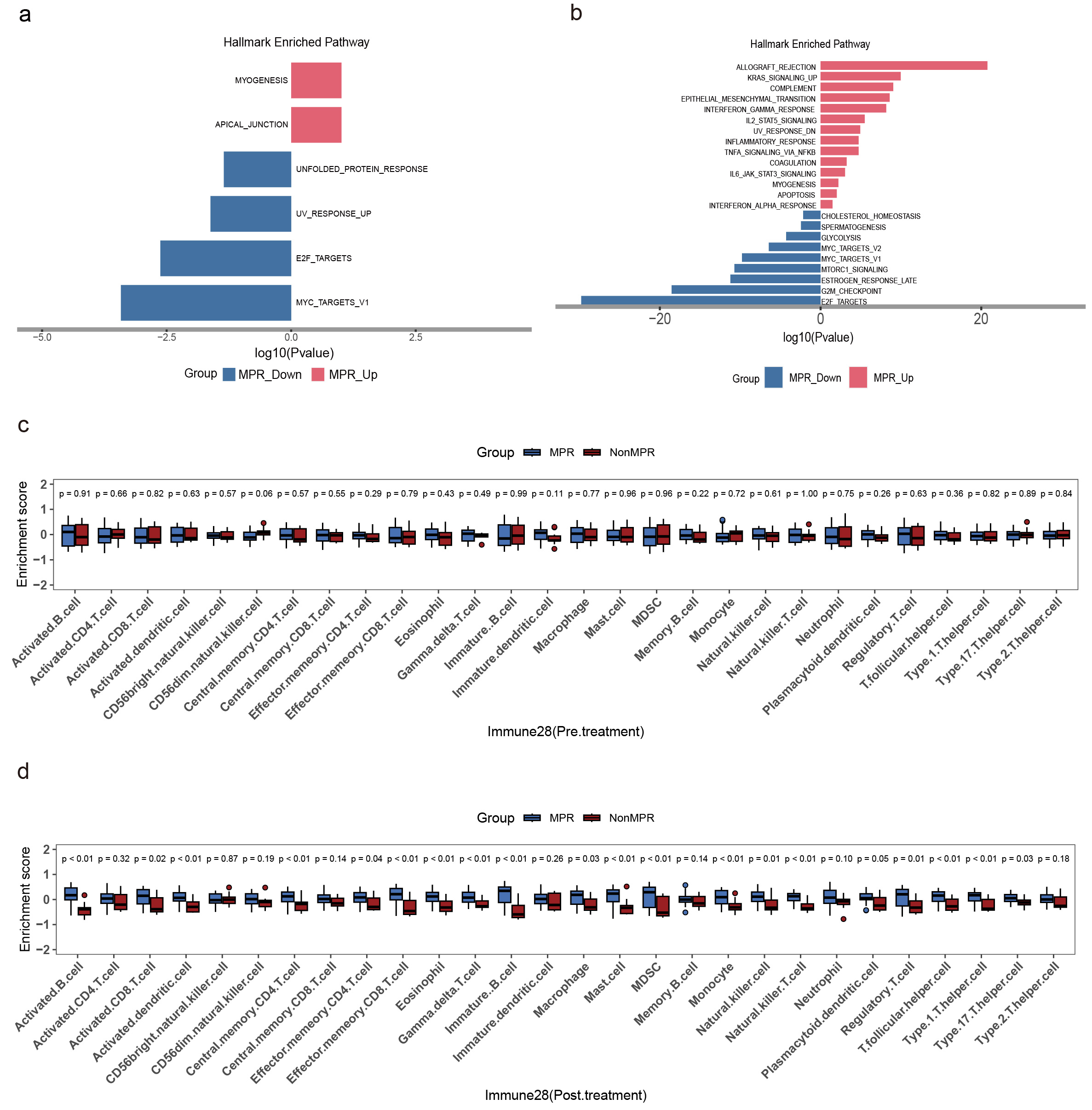

Supplement: Supplementary Figure 1 — Differential gene signaling pathway enrichment and tumor microenvironment differences between MPR and non MPR groups. (a) KEGG enrichment analysis of differentially expressed genes of MPR vs non-MPR samples pre-NCI. (b) KEGG enrichment analysis of differentially expressed genes of MPR vs non-MPR samples post-NCI. (c) Differences in enrichment of immune cells between MPR and non-MPR groups pre-NCI. (d) Differences in enrichment of immune cells between MPR and non-MPR groups post-NCI. [file Image1.jpeg]

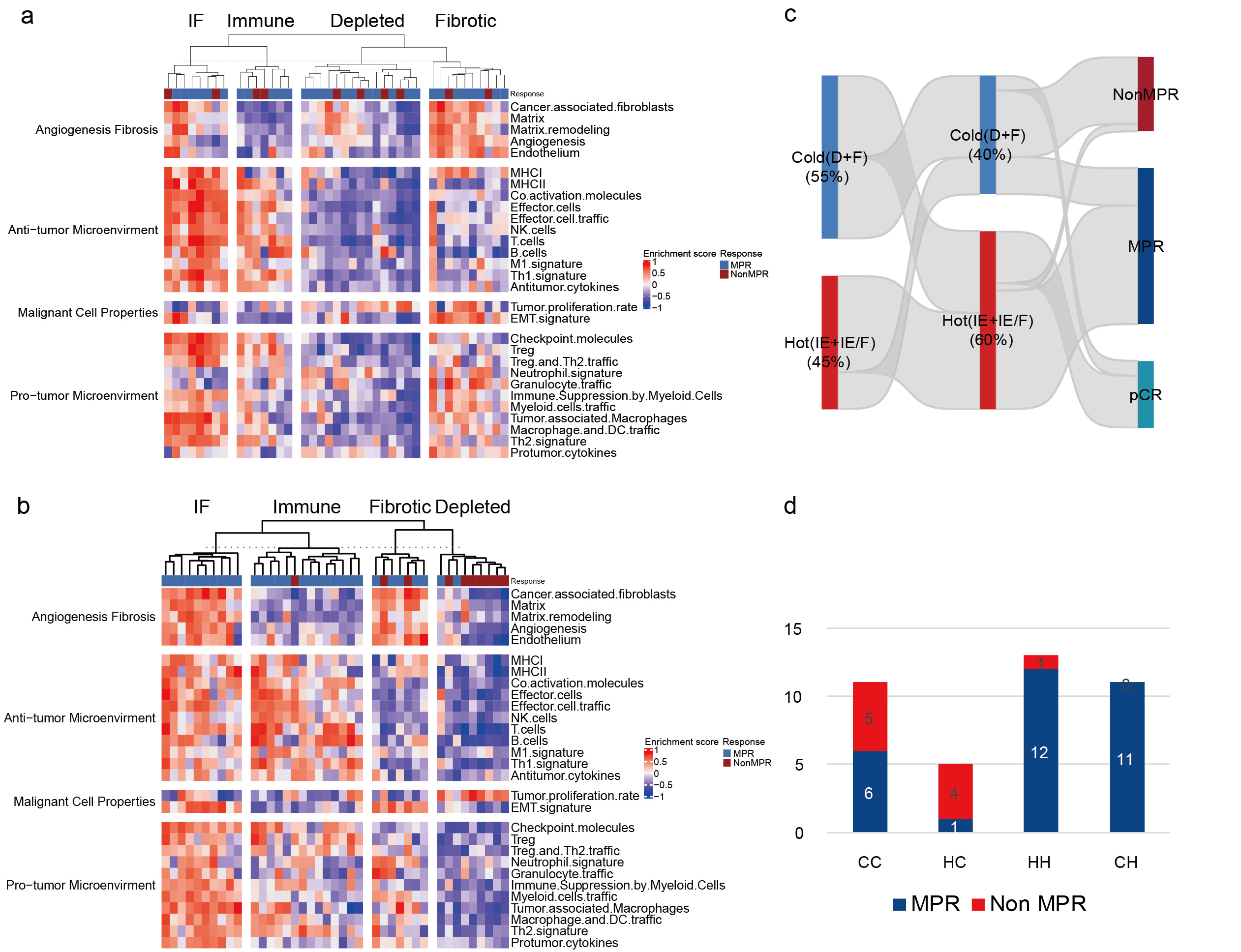

Supplement: Supplementary Figure 2 — Distribution of “cold” and “hot” tumors pre- and post-NCI treatment between MPR and non MPR groups. (a) Pre-treatment, clustering analysis of the immune microenvironment based on previously published literature was performed to define “hot” and “cold” tumors. (b) Post-treatment, clustering analysis of the immune microenvironment based on previously published literature was performed to define “hot” and “cold” tumors. (c) Sankey plot demonstrating the dynamics of “hot” and “cold” tumors between different treatment responses pre- and post-treatment. (d) Distribution of MPR and non-MPR samples across treatment effects. Based on the difference between hot and cold tumors pre- and post-NCI treatment, we classified treatment effects into 4 types: tumors transitioning from “hot” to “cold” (HC); maintaining “cold” tumor (CC); tumors transitioning from “cold” to “hot” (CH); and maintaining “hot” tumor (HH). [file Image2.jpeg]

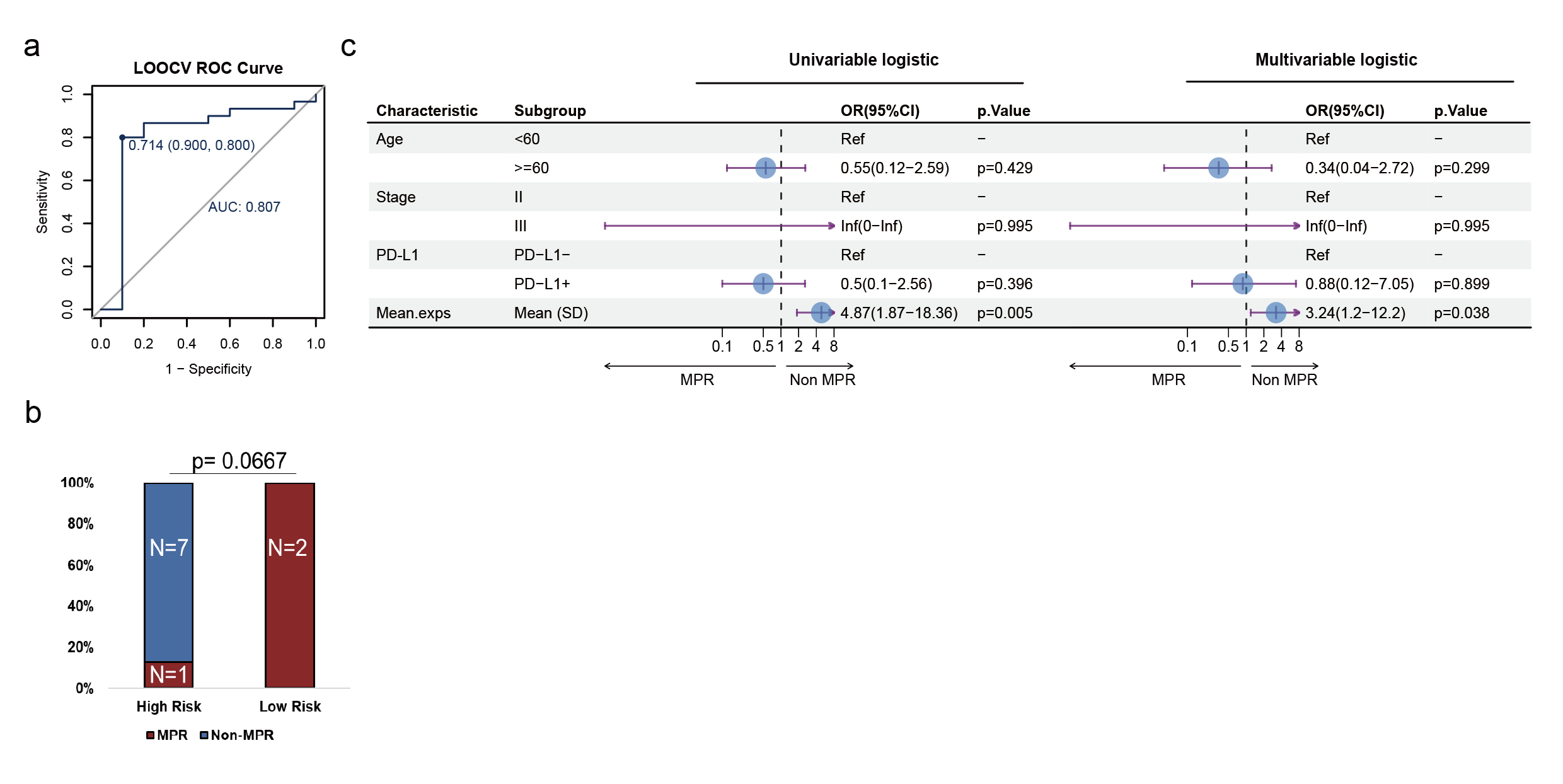

Supplement: Supplementary Figure 3 — Seven-gene expression performance verification. (a) Leave-one-out cross-validation (LOOCV) of the 7-gene score for predicting MPR; ROC curve with AUC reported. (b) Validation of the 7-gene signature in an independent neoadjuvant LUSC biopsy cohort (GSE207422; Junjie Hu et al., Genome Medicine, 2023). Patients (n=10) were stratified into High-risk and Low-risk groups using the optimal cutoff of the 7-gene score. (c) Multivariable logistic regression of the 7-gene score for MPR prediction (adjusted for stage, age, and PD-L1 status); forest plot shows adjusted odds ratios (95% CIs) and p-values. [file Image3.jpeg]

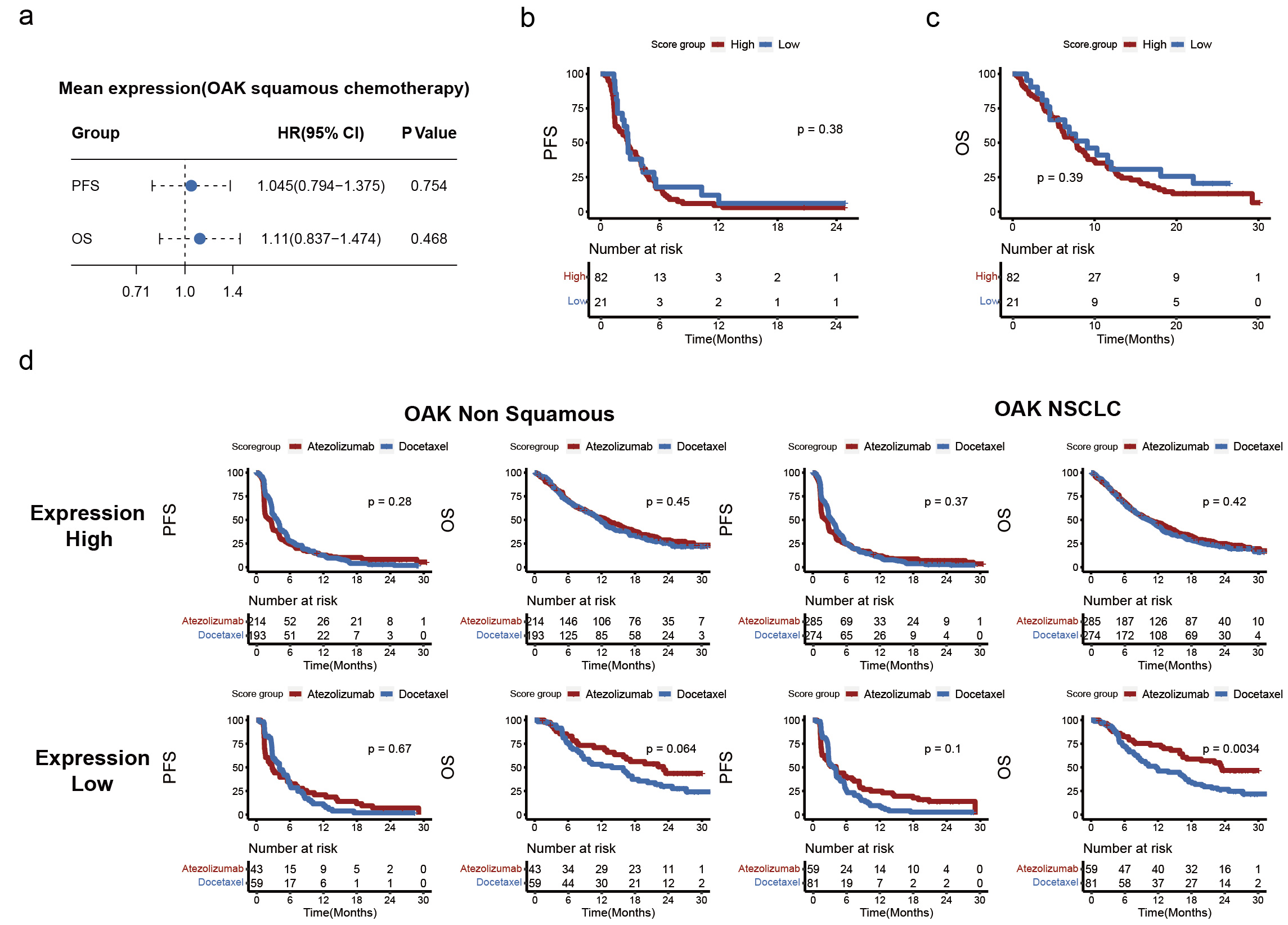

Supplement: Supplementary Figure 4 — Validation of external data showing association of seven genes expression levels with chemotherapy PFS and OS in LUSC. (a) Based on the OAK database, the correlation between the expression of seven genes and OS and PFS in LUSC patients receiving chemotherapy was analyzed by univariate COX regression. (b) In LUSC patients receiving chemotherapy, Kaplan-Meier survival curves showed OS and PFS in patients with high and low gene expression. (c) Based on OAK database, PFS and OS were compared by Kaplan-Meier survival analysis between immunotherapy and chemotherapy groups in non-LUSC patients and whole NSCLC group. [file Image4.jpeg]

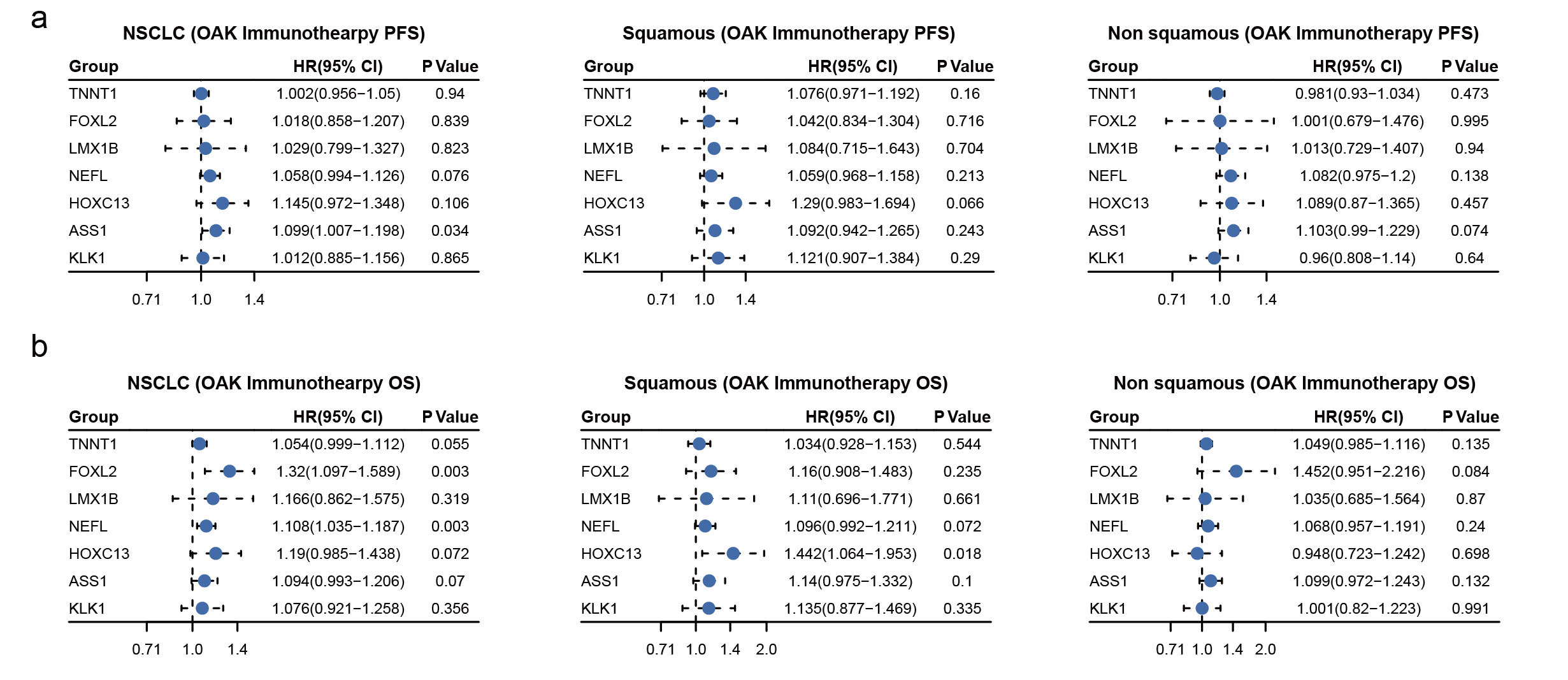

Supplement: Supplementary Figure 5 — HOXC13 expression is associated with poor outcome of LUSC immunotherapy. (a) Expression of seven genes in the OAK database correlates with PFS in NSCLC, LUSC, and LUAD patients by univariate COX regression analysis. (b) Expression of seven genes in the OAK database correlates with OS in NSCLC, LUSC, and LUAD patients by univariate COX regression analysis. [file Image5.jpeg]
