# Supplementary material for: A 7-gene expression signature predicts immune microenvironment remodeling and neoadjuvant chemo-immunotherapy response in lung squamous cell carcinoma
Source: Front Immunol. 2026 Feb 26;17:1696792. doi: 10.3389/fimmu.2026.1696792 (PMC12979163; doi:10.3389/fimmu.2026.1696792)
Supplement: Supplementary file 6 [file DataSheet1.pdf]

Supplementary Table1: Single cell subtypes		
	Subtypes	eference PMID
ECs	ECs_C1_ESM1	PMID: 32561858.
	ECs_C2_ACKR1	
	ECs_C3_CA4	
	ECs_C4_FBLN5	
	ECs_C5_PROX1	
	ECs_Capillary_C3_NEC1	
	ECs_Capillary_C3_NEC2	
	ECs_Capillary_C3_NEC3	
	ECs_Capillary_C3_NEC4	
	ECs_Capillary_C3_TEC	
Fibroblast	Fibroblast_C10_COMP	
	Fibroblast_C11_SERPINE1	
	Fibroblast_C7_MYH11	
	Fibroblast_C8_RGS5	
	Fibroblast_C9_CFD	
DC	DC_C1_CLEC9A	
	DC_C2_CLEC10A	
	DC_C3_CCR7	
	DC_C4_LILRA4	
	DC_C5_CD207	
T/NK	T_NK_C1_CD8_HAVCR2	
	T_NK_C2_CD8_GZMK	
	T_NK_C3_CD8_ZNF683	
	T_NK_C4_CD8_CX3CR1	
	T_NK_C5_CD4_CCR7	
	T_NK_C6_CD4_GZMA	
	T_NK_C7_CD4_CXCL13	
	T_NK_C8_CD4_FOXP3	
	T_NK_C9_NK_FGFBP2	
	T_NK_C10_NK_XCL1	
B cell	Bcell_C1_CD27-/IGHD+	
	Bcell_C2_CD27+/IGHM+	
	Bcell_C3_CD27+/IGHM-	
	Bcell_C4_CD27+/CD38+	
	Bcell_C5_IGHG1+/PRDM1-high	
	Bcell_C6_IGHG1+/PRDM1-low	
	Bcell_C7_IGHA1+/PRDM1-high	
	Bcell_C8_IGHA1+/PRDM1-low	
Myeloid	Myeloid_C1_CD14	
	Myeloid_C2_CD16	
	Myeloid_C3_CCR2	
	Myeloid_C4_CCL2	
	Myeloid_C5_CCL18	

Myeloid	Myeloid_C6_MMP9	
	Myeloid_C7_CX3CR1	
	Myeloid_C8_PPARG	
	Myeloid_C9_LYVE1	
	Myeloid_C10_FCGR3B	
Mast cell	Mast_C1_CPA3	
	Mast_C3_ICAM1	
Alveolar	Alveolar_C1_AGER	
	Alveolar_C2_SFTPC	
	Alveolar_C3_CHI3L1	
	Alveolar_C4_SCGB1A1	
	Alveolar_C5_KRT5	
DC	cDC1.specific	PMID: 33545035.
	cDC2.specific	
	cDC2_CXCR4hi	
	cDC2_CD1A	
	cDC2_IL1B	
	cDC2_FCN1	
	cDC2_ISG15	
	cDC2_CXCL9	
	ActivatedDC	
	MigratoryDC	
Fiborblast/Endothelium	Fibroblast_c1_RGS5	PMID: 36333338.
	Fibroblast_c2_COL1A1	
	Fibroblast_c3_DCN	
	Fibroblast_c4_CFD	
	Fibroblast_c5_DCN	
	Fibroblast_c6_RGS5	
	Fibroblast_c7_S100B	
	Fibroblast_c8_RGS5	
	Endothelium_c25_ACKR1	
	Endothelium_c26_FCN3	
	Endothelium_c27_FABP5	
	Endothelium_c28_PLVAP	
NK	CD56dimCD16hi-c1-IL32	PMID: 37607536.
	CD56dimCD16hi-c2-CX3CR1	
	CD56dimCD16hi-c3-ZNF90	
	CD56dimCD16hi-c4-NFKBIA	
	CD56dimCD16hi-c5-MKI67	
	CD56dimCD16hi-c6-DNAJB1	
	CD56dimCD16hi-c7-NR4A3	
	CD56dimCD16hi-c8-KLRC2	
	CD56brightCD16lo-c1-GZMH	
	CD56brightCD16lo-c2-IL7R-RGS1lo	
	CD56brightCD16lo-c3-CCL3	

	CD56brightCD16lo-c4-IL7R	
	CD56brightCD16lo-c5-CREM	
	CD56brightCD16hi	
CD4 T/CD8 T	CD4.c01(Tn)	PMID: 34914499.
	CD4.c02(CXCR5+ pre-Tfh)	
	CD4.c03(ADSL+ Tn)	
	CD4.c04(IL7R- Tn)	
	CD4.c05(TNF+ T)	
	CD4.c06(AREG+ Tm)	
	CD4.c07(TIMP1+ Tm)	
	CD4.c08(CREM+ Tm)	
	CD4.c09(CCL5+ Tm)	
	CD4.c10(CAPG+ Tm)	
	CD4.c11(CAPG+CREM- Tm)	
	CD4.c12(GZMK+ Tem)	
	CD4.c13(Temra)	
	CD4.c14(CCR6+ Th17)	
	CD4.c15(IL26+ Th17)	
	CD4.c16(IL21+ Tfh)	
	CD4.c17(IFNG+ Tfh/Th1)	
	CD4.c18(TNFRSF9- Treg)	
	CD4.c19(S1PR1+ Treg)	
	CD4.c20(TNFRSF9+ Treg)	
	CD4.c21(ISG+ Treg)	
	CD4.c22(ISG+ Th)	
	CD4.c23(NME1+CCR4- T)	
	CD4.c24(NME1+CCR4+ T)	
	CD8.c01(Tn)	
	CD8.c02(IL7R+ Tm)	
	CD8.c03(uncharacterized)	
	CD8.c04(ZNF683+CXCR6- Tm)	
	CD8.c05(GZMK+ early Tem)	
	CD8.c06(GZMK+ Tem)	
	CD8.c07(Temra)	
	CD8.c08(KIR+EOMES+ NK-like)	
	CD8.c09(KIR+TXK+ NK-like)	
	CD8.c10(ZNF683+CXCR6+ Trm)	
	CD8.c11(GZMK+ Tex)	
	CD8.c12(terminal Tex)	
	CD8.c13(OXPHOS- Tex)	
	CD8.c14(TCF7+ Tex)	
	CD8.c15(ISG+ CD8+ T)	
	CD8.c16(Tc17)	
	CD8.c17(NME1+ T)	
	TRN	PMID:

Neutrophil	NAN	PMID: 36368318.
	TAN	
